# Targeted allele frequency tuning in breeding populations via alternative optimum contribution selection

**DOI:** 10.1093/genetics/iyag102

**Published:** 2026-04-24

**Authors:** Tobias A M Niehoff, Marije J Steensma, Harmen P Doekes, Mario P L Calus, Bart J Ducro, Matias F Schrauf

**Affiliations:** Animal Breeding and Genomics, Wageningen University & Research, Droevendaalsesteeg 1, P.O. Box 338, Wageningen 6700AH, The Netherlands; Animal Breeding and Genomics, Wageningen University & Research, Droevendaalsesteeg 1, P.O. Box 338, Wageningen 6700AH, The Netherlands; Koninklijke Vereniging “Het Friesch Paarden-Stamboek”, Lavendelheide 13, Drachten 9202DP, The Netherlands; Animal Breeding and Genomics, Wageningen University & Research, Droevendaalsesteeg 1, P.O. Box 338, Wageningen 6700AH, The Netherlands; Animal Breeding and Genomics, Wageningen University & Research, Droevendaalsesteeg 1, P.O. Box 338, Wageningen 6700AH, The Netherlands; Animal Breeding and Genomics, Wageningen University & Research, Droevendaalsesteeg 1, P.O. Box 338, Wageningen 6700AH, The Netherlands; Animal Breeding and Genomics, Wageningen University & Research, Droevendaalsesteeg 1, P.O. Box 338, Wageningen 6700AH, The Netherlands

**Keywords:** relationship matrix, genomic relationship, lethal alleles, allele frequency, optimum contribution selection, Friesian horses

## Abstract

When managing the rate of genomic inbreeding using genomic relationship matrices, allele frequencies tend to be moved toward the frequencies used for centering genotypes in those matrices. Here, we explore if this behavior could be exploited to purposefully change the frequency of alleles. We test the method in a simulated toy example and in the Friesian horse population to purge deleterious alleles as a case study. We show that the average entry of the numerator matrix from VanRaden's method 1 $\left(\bar{ZZ^{\prime }}\right)$ equals 4 times the average squared difference between the populations’ allele frequency (AF) and the used centering allele frequency (CAF) $\left(\bar{ZZ^{\prime }}=4\overset{\mathrm{nsnp}}{\underset{k=1}{\sum }}\;{\left(AF_{k}-\mathrm{CA}F_{k}\right)}^{2}\right)$. By setting the centering frequency to the desired AF and subsequently optimizing contributions to minimize the average matrix entry, the population's AF is shifted to the desired frequency. We call this novel selection method using a matrix: “TAFT” (targeted allele frequency tuning), and use it with optimum contribution selection (OCS) in this study (TAFT-OCS). TAFT outperformed index methods for target frequencies between 0% and 100%. The index methods showed different behavior compared to TAFT by exerting different selection pressure on loci depending on the difference to the target frequency. Frequencies of all 12 deleterious alleles could be reduced within ∼6 generations in the Friesian horse population so that no affected individuals remained. At the same time, genetic gain of 3.7 cm withers height (1.11 gSD) per generation was made and the realized pedigree inbreeding rate kept below 0.5%. We propose TAFT-OCS as a novel method to directly change allele frequencies to any desired value which is particularly beneficial if intermediate allele frequencies are desired or genetic diversity should be maintained. TAFT shifts the focus of selection from individuals to alleles. It only requires to set a desired target frequency and a minimum and maximum allowed frequency if needed.

## Introduction

Animal breeding programs typically aim to genetically improve a variety of traits of interest, while limiting the rate of inbreeding. While many traits of interest are polygenic, some are influenced by major QTLs with extraordinary large effect sizes. Examples are DGAT1 for milk yield and fat composition in taurine dairy cattle (Grisart et al. 2004), the Booroola gene affecting fecundity in Merino sheep (Fabre et al. 2003; Reader et al. 2012), and LCORL affecting withers height in German warmblood horses (Reich et al. 2024). Apart from polygenic traits, monogenic traits (influenced by a single gene) and oligogenic traits (influenced by a few genes) exist. Examples include polledness in Holstein dairy cattle (Rothammer et al. 2014), the introgressed *SLICK* gene for improved thermotolerance in Holstein dairy cattle (Zayas et al. 2025), color and morphological traits in betta fighting fish (Zhang et al. 2022), and semen traits in pigs (Marques et al. 2018). While these traits are important, assigning an economic value may not always be straightforward. Lastly, deleterious or even lethal alleles segregate in animal populations (Agerholm and Peperkamp 2007; Ducro et al. 2015; Leegwater et al. 2016; Donner et al. 2018; Derks and Steensma 2021; van den Berg et al. 2024). These genes are not necessarily included in a selection index yet breeders would like to control their frequency, most often with the intention to purge them. All these cases have in common that the effect of the allele is known. Therefore, breeders can aim to decrease or increase the allele frequency (AF) or even aim for a particular “optimal” frequency. However, constructing a good strategy to achieve these desired frequencies is not straightforward.

Previous studies have investigated several strategies to move alleles at important loci toward fixation, in most cases with the aim to purge (lethal) deleterious variants. For example, de Cara et al. (2013) investigated the use of inbred matings, i.e. matings between close relatives, to expose recessive deleterious variants to natural selection and facilitate purging. However, no clear advantage of matings between related animals with regard to fitness and overall neutral diversity was found. Sonesson et al. (2003) reduced the prevalence of a genetic disease by selecting for animals with a low allele count for the disease in the simulated single-gene model by treating allele count as the animal's estimated breeding value (EBV) and managing genome-wide inbreeding with optimum contribution selection (OCS) (Meuwissen 1997). Also within the framework of OCS, Fernández et al. (2006) and Engelsma et al. (2014) explored strategies to select a group of animals whose AF matches a predefined frequency as much as possible. Their work is focused on a single locus and is motivated by selecting animals such that particular alleles are well represented in a gene bank. Hjortø et al. (2021) explored the use of preselection against carriers of a single lethal recessive allele. While preselection led to faster purging of the variant compared to natural selection, the authors acknowledged that culling carriers may not be the best strategy when many alleles or alleles with a high frequency are to be purged. Since (almost) no animal is free from unfavorable alleles, culling all carriers would create a population bottleneck (Georges et al. 2019), reducing genetic variation and increasing the chance novel deleterious alleles drifting to high frequencies. Instead of culling carriers, Georges et al. (2019) suggested to infer the cost of genetic defects and to include the defects in a selection index. This would lead to a more gradual frequency reduction of the variants as in Sonesson et al. (2003).

Other studies have also investigated strategies to maintain diversity (i.e. keep current allele frequencies) or maximize diversity (i.e. move them toward 0.5) in specific genomic regions. For example, Roughsedge et al. (2008) used OCS to maintain diversity around a locus with large effect (major QTL) on the breeding goal trait during selection. To limit the loss of diversity around the QTL, they used a relationship matrix that was not purely based on the pedigree (as in traditional OCS), but also based on markers around the QTL. Using semidefinite programming (SDP), Gómez-Romano et al. (2016) extended traditional OCS to also maintain or even increase heterozygosity in specific regions of the genome for which high diversity is deemed desirable, such as the major histocompatibility complex (MHC). This is done by adding an additional constraint for each set of genomic regions in the OCS formulation, with a desired maximum rate of inbreeding per region. Doekes (2020, p. 168) furthermore suggested to extend this SDP approach to move or keep alleles (e.g. polled allele in cattle) to a predefined target frequency. However, this would imply adding an additional constraint for every locus of interest, or build an index and add a constraint to this index.

Rather than managing specific regions, the optimal use of relationship matrices in OCS to limit the rate of genome-wide inbreeding has been tested (Sonesson et al. 2012; Henryon et al. 2019; Meuwissen et al. 2020; Morales-González et al. 2021; Gautason et al. 2023). With only pedigree information available, the pedigree relationship matrix is the only matrix to control the increase of average kinships of the animals in the breeding population (hereafter referred to as “inbreeding rate”). However, if genotypes of animals are available, a genomic relationship matrix (GRM) can be used to control the rate of inbreeding. Genomic inbreeding control is expected to outperform pedigree-based control, because genomic relationships also capture Mendelian sampling (Daetwyler et al. 2007). However, simulation studies have shown that the use of genomic relationships is not always better than the use of pedigree-based relationships (Sonesson et al. 2012; Henryon et al. 2019) and that it depends on the GRM that is used (Meuwissen et al. 2020). This is because multiple methods to construct GRMs exist and these GRMs are not identical. An important aspect related to this issue, as recognized by Meuwissen et al. (2020) and Gautason et al. (2023), is whether genomic relationships, and thus genomic inbreeding, should be based on the homozygosity at the observed loci or on the change of AF from some reference, usually the base generation. For the first, we get homozygosity-based inbreeding:


(1) $$F_{\mathrm{hom}}=\underset{k}{\sum }\frac{\;p{\left(\mathrm{hom}\right)}_{t,k}}{N_{\mathrm{SNP}}}=\underset{k}{\sum }\frac{1-p{\left(\mathrm{het}\right)}_{t,k}}{N_{\mathrm{SNP}}}\approx \underset{k}{\sum }\frac{1-2p_{t,k}(1-p_{t,k})}{N_{\mathrm{SNP}}}$$

where the approximation is valid if genotype frequencies are in Hardy–Weinberg equilibrium. While for the second, we get “drift-based inbreeding”:


(2) $$F_{\mathrm{drift}}=\left(\underset{k}{\sum }\frac{{\left(\;p_{t,k}-p_{t=0,k}\right)}^{2}}{\left(\;p_{t=0,k}(1-p_{t=0,k})\right)}\right)\times \frac{1}{N_{\mathrm{SNP}}}$$

When managing diversity with a GRM based on VanRaden (2008) method 1 (VR1), allele frequencies are “pulled” toward those used as reference for centering (centering allele frequency = CAF) in the calculation of relationships, as observed in simulation studies (Meuwissen et al. 2020; Morales-González et al. 2021; Gautason et al. 2023). This behavior was the motivation for our study. In the calculation of VR1 relationships between 2 individuals $\left(2f_{i,j}\right)$,


(3) $$2f_{i,j}=\left(\underset{k}{\sum }\;(AD_{i,k}-2\mathrm{CA}F_{k})(AD_{\;j,k}-2\mathrm{CA}F_{k})\right)\times \frac{1}{\sum_{k}\;2\mathrm{CA}F_{k}(1-\mathrm{CA}F_{k})}$$

one may notice that deviations from CAF are more “punished,” i.e. result in higher relationships, the larger the differences between allele dosages of individuals $\left(AD_{i,k},AD_{\;j,k}\right)$ and the CAF. Note that for this we use the notation for animal's genotypes as in Hayes et al. (2009) with “0” coding for homozygous genotype states for the reference allele, “1” coding for heterozygous loci, and “2” coding for homozygous states for the alternative allele. If two individuals are homozygous for the reference allele, which is typically chosen to be the allele with the higher observed frequency, they would appear to be less related than if they were homozygous for the alternative allele, that is, as long as the observed frequency of the reference allele is higher than 50% and is used as the CAF. If the CAF for all SNPs are 50%, VR1 results in relationships that are perfectly correlated to the identity-by-state (IBS) relationships ((VR1 + 2)/2 = IBS), i.e. reflect expected homozygosity-based relationships at the genotyped SNPs. For completeness, note that IBS relationships are equivalent to “similarities” as defined by Eding and Meuwissen (2001), which was shown by Eynard et al. (2015) (see their Additional file 1) (the term similarity” was more frequently used before the publication of VanRaden (2008)). Optimization of VR1 with CAF of 50% consequently reflects minimizing homozygosity-based inbreeding (Eq. 1) and tends to move allele frequencies of genotyped loci to 50%. Optimization of VR1 with CAF as those observed in the base generation aims to reduce drift-based inbreeding and tends to maintain allele frequencies at base frequencies. Note that “homozygosity-based” and “drift-based” inbreeding strictly only refer to inbreeding and relationships at the genotyped (and managed) loci but do not refer to inbreeding and relationships at ungenotyped loci.

Whereas CAFs are typically those from the base population or set to 50%, any other value could also be used. The findings of Gautason et al. (2023) suggest that targeted changes of AF could be achieved with a GRM in which the CAF are set to the desired frequencies of the alleles. In other words, we could construct a GRM including e.g. only loci with deleterious mutations, set the frequencies for centering to 0%, and then select a set of animals whose average entry on that GRM is minimal. That should drive the frequencies of these deleterious mutations to 0%. Conversely, setting the CAF to 100% should eventually lead to fixation.

This study has three goals. First, we present equations and explain why changing the CAF for centering and subsequent selection of a group of animals that minimizes the average entry of the numerator matrix of the relationship matrix results in (close to) desired allele frequencies. Second, we test its use in simulated toy selection schemes to understand the behavior of the method. Third, we evaluate the use of a GRM with modified CAF in a more realistic scenario. For this scenario, we assume population parameters of the Friesian horse breed and simulate an idealized management scheme, including purging of deleterious alleles, maintaining diversity at the MHC locus, limiting genome-wide inbreeding, and making genetic progress for a breeding goal trait.

## Methods

### Theory

In this study, we explore three approaches to change allele frequencies: a novel matrix-based method and two selection index approaches. The matrix method will be called targeted allele frequency tuning (TAFT), and selections will be done via OCS (TAFT-OCS). This method exploits properties of VanRaden's method 1 for calculating genomic relationships, as will be explained in this section.

The numerator of VR1 relationship matrices is $ZZ^{\prime }$ with Z calculated as Z=M−P, where M is a matrix of dimensions *n* (number of individuals) by *m* (number of loci) that stores the allele dosage (0, 1, 2) for every individual at loci of interest according to Hayes et al. (2009). In the publication of VanRaden (2008), the notation was −1, 0, 1, but this notation is not used in this manuscript. P is an n×m matrix in which every column contains the CAF multiplied by 2 for the respective locus. Thus, elements of Z are twice the deviation of an animal's AF (0, 0.5, 1) from twice the CAF. Consequently, elements of $ZZ^{\prime }$ store sums of products of the allele dosage deviations from twice the CAF. More precisely, off-diagonal elements store the sum of the product of the individual deviations of two animals. The diagonal elements indicate the sum of the squares of an individual's deviations. The latter is identical to four times the sum of an individual's AF deviation squared.

The average element of $ZZ^{\prime }$ is 4 times the sum of the squares of the deviation of the AF observed in the set of individuals from the CAF:


(4) $$\bar{ZZ^{\prime }}=4\overset{N_{\mathrm{SNP}}}{\underset{k=1}{\sum }}\;(AF_{k}-\mathrm{CA}F_{k}{)}^{2}$$

**Fig. 1.**
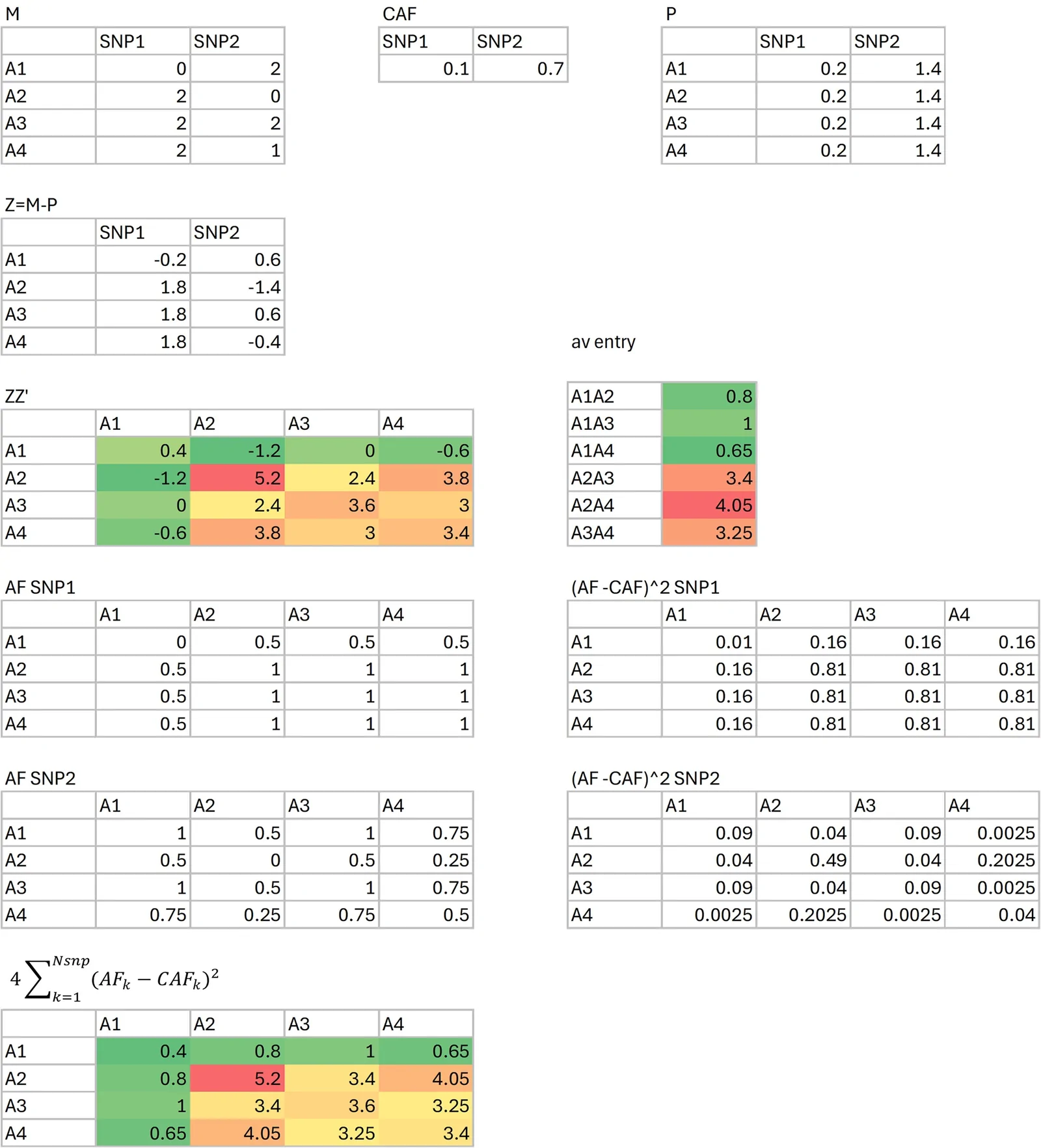
Exemplary calculation of the $ZZ^{\prime }$ matrix and similarity to squared AF deviation.

Note that the left-hand side of the equation is an average since Z contains deviations for all SNPs, and the right-hand side is a sum. A proof for this is shown in the Appendix. Figure 1 gives an example for 2 SNPs and 4 individuals. The diagonal entries in $ZZ^{\prime }$ are identical to the diagonal entries in the matrix storing 4 times the sum of the squared AF deviations. The off-diagonal elements of the matrix storing 4 times the sum of the squared AF deviations are identical to the average of the 4 cells in $ZZ^{\prime }$ belonging to the 2 individuals in question. For example, the average AF of the group consisting of animals A2 and A3 is 100% for SNP1 and 50% for SNP2. Four times the sum of the squared deviations is 3.4 (=4[(1 − 0.1)^2^ + (0.5 − 0.7)^2^]). This is identical to the average of the $ZZ^{\prime }\;$ restricted to cells belonging to A2 and A3 ((5.2 + 2.4 + 2.4 + 3.6)/4 = 3.4).

**Fig. 2.**
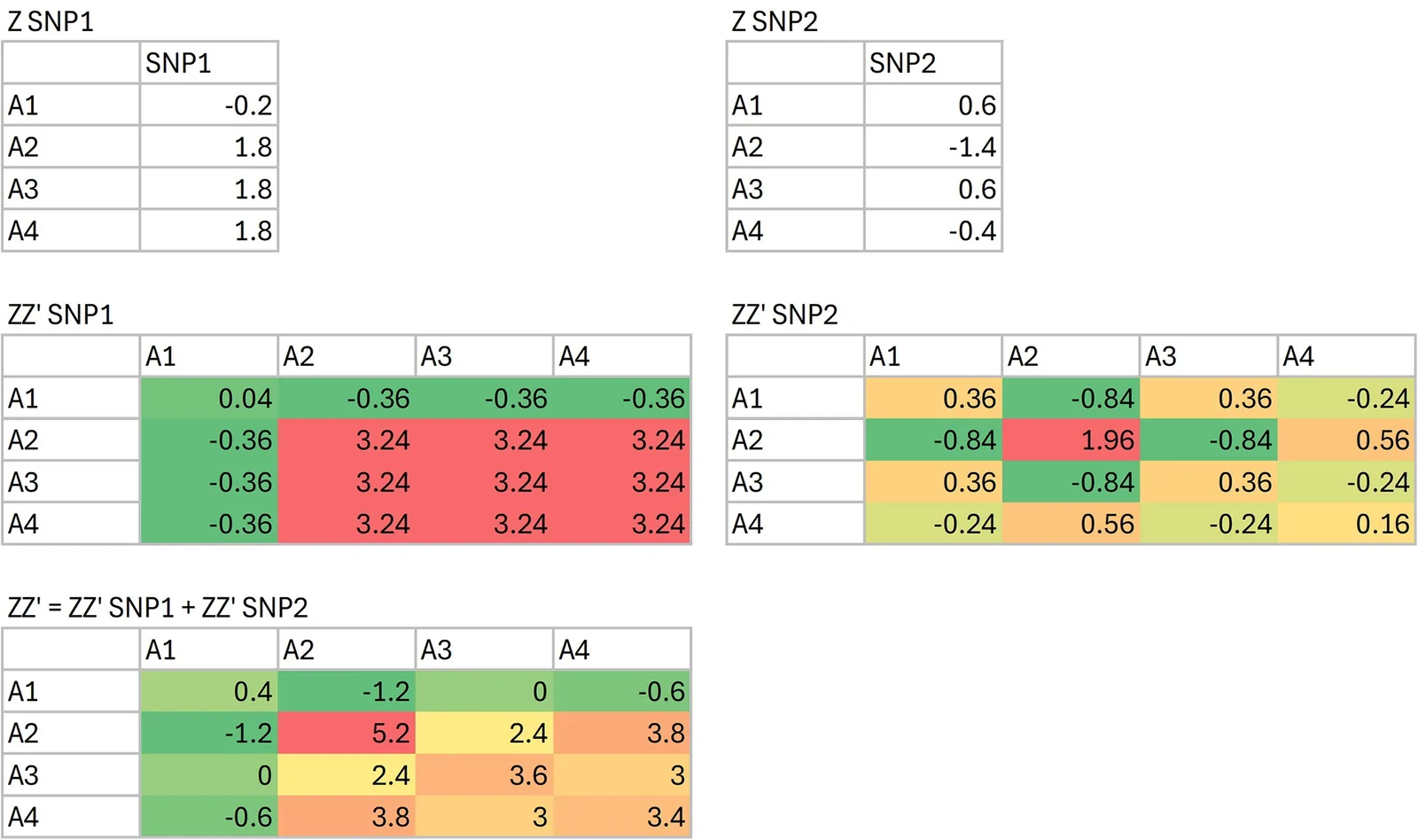
The total $ZZ^{\prime }$ matrix is the sum of $ZZ^{\prime }$ matrices of all loci.

For better intuition, consider that the $ZZ^{\prime }$ matrix based on all SNPs can also be understood as the sum of $ZZ^{\prime }$ matrices constructed for each marker (Fig. 2). $ZZ^{\prime }$ matrices constructed for each marker are constructed on the Z matrix for each marker which is just an individual column of the Z matrix constructed for all markers (compare Z matrices in Figs. 1 and 2).

**Fig. 3.**
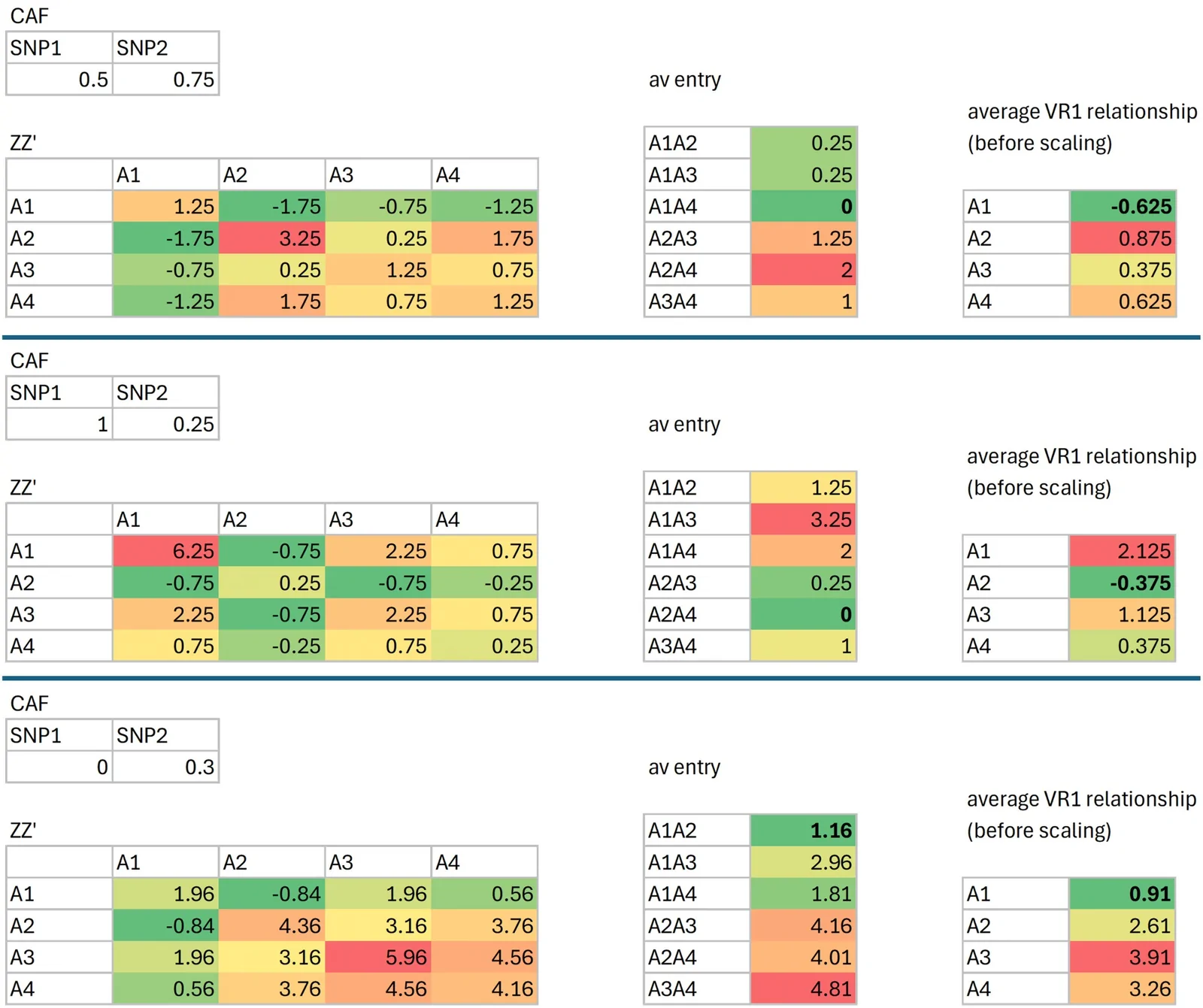
Matrix $ZZ^{\prime }$ calculated with different allele frequencies for centering. The average entry value of all groups of two individuals is indicated as well as the average relationship (before scaling) of animals to all four animals. The lowest average entry value is indicated in bold. Values are color formatted so that lowest values are green and highest values are red.

The most important realization is that different allele frequencies used for centering would result in different sets of individuals being selected if the objective is to minimize the average “relationship” (reduce the sum of squared deviations). To construct a GRM according to VR1, $ZZ^{\prime }$ is scaled by dividing the matrix with the sum of 2pq of all loci. This scales the values in the numerator $\left(ZZ^{\prime }\right)$ but does not change their ranking. In other words, the scaling factor does not change the ranking or the relative differences between relationship values. Thus, it matters what frequencies are used for centering as it would result in reranking of relationships and thus in different selection decisions for inbreeding management. Consider the examples given in Fig. 3: the same individuals as in Fig. 1 are used but now $ZZ^{\prime }$ is constructed with different CAF. If we had to select a group of 2 individuals in this small example based on minimizing the squared AF deviation (the numerator used for constructing a GRM), we would make different decisions depending on the CAF.

This relationship between average matrix entries and squared AF deviations enables targeted management of AF. As in the example in Fig. 3, a group of individuals that would result in the lowest average value could be selected. The AF of the offspring of these animals would then have the smallest average squared AF deviation from the desired frequencies. Consequently, animals with the lowest average “relationship” on such a matrix could be used to change the allele frequencies toward desired values, analogously with mean kinship selection to control the inbreeding rate. A more elaborate solution would be to fit such a matrix in OCS software to find optimal contributions $\left(c_{t}\right)$ of animals in the current generation *t* that minimize the average matrix entry:


(5) $$\bar{{\left(AF_{k,t+1}-\mathrm{CA}F_{k}\right)}^{2}}={c^{\prime }}_{t}\frac{ZZ^{\prime }}{4\times N_{\mathrm{SNP}}}c_{t}$$

$ZZ^{\prime }$ is divided by 4 times the number of SNPs in Eq. 5 for better interpretability so that the average squared deviation per SNP is expressed. Since this just scales the matrix entries, it would not change relative differences of relationships and thus does not result in different selection decisions or rankings.

### Toy example

#### Description of the population

We tested the performance of selecting with a $ZZ^{\prime }$ matrix with CAF set to the desired frequencies: TAFT. A population of 100 individuals of a species with a genome of 25 chromosomes each of length 1 Morgan was simulated using the R package MoBPS version 1.11.90 (Pook et al. 2020) in R version 4.4.1. That version can be downloaded from Torsten Pook's GitHub page (https://github.com/tpook92/MoBPS). The base population was constructed based on default parameters in MoBPS and did not show linkage disequilibrium or family relationships. Selection was done with OCS each generation starting with the base with the R package optiSel version 2.0.9 (Wellmann 2019). The OCS problem was stated such that the pedigree inbreeding level would not increase by more than 1% (as percentage point) per generation while the average entry of $ZZ^{\prime }$ weighted by the contributions was minimized. The inbreeding rate was managed to reduce the chance of accidentally losing beneficial alleles due to drift. The pedigree relationship matrix was used for simplicity. To generate a new candidate, a sire and a dam were chosen at random with probabilities according to the genetic contributions obtained with optiSel as in Meuwissen (1997). If optiSel returned an invalid solution or if the solution was not at the boundary of the solution space, a different solver was tried until a valid boundary solution was found. If none of the solvers yielded a boundary solution, any valid solution was used. Our default solver was “slsqp” as it is the most generalist solver. The other tried solvers were “cccp” and “alabama.” Solver slsqp does gradient-based optimization with sequential least-squares quadratic programming as implemented in function slsqp() of the R package nloptr (author Hans W. Borchers in Johnson 2008). Solver cccp is from the R package cccp (Pfaff 2025) for solving cone constrained convex programs, and solver alabama is from the R package alabama (Varadhan 2023) which solves the optimization problem by augmented lagrangian minimization. All solvers were used as described in the manual of optiSel.

#### Scenarios of targeted allele frequencies

Before the first round of selection, five random loci were selected that showed alleles with frequencies of 95%, 75%, 50%, 25%, and 5%. These did not affect fitness, i.e. natural selection would not change their frequency. Only these five loci were used to construct the $ZZ^{\prime }$ matrix. Apart from trying to purge these alleles by artificial selection, i.e. change their frequencies to 0%, we experimented with other goals. These goals were obtaining the same frequency at all of these SNPs (80% or 70%) as well as changing—and then maintaining—them at SNP-specific target frequencies (10%, 20%, 30%, 40%, and 50%, respectively). Lastly, we also increased the number of loci with alleles to purge from 5 to 10, 50, and 100.

#### Approaches to change allele frequencies

In total, we used three approaches to change allele frequencies at the loci of interest. In the first approach, the CAFs were adjusted in the $ZZ^{\prime }$ matrix as explained before, and we called this method TAFT-OCS. The second and third approaches use selection indices, and their motivation is explained hereafter.

The second approach was the use of a selection index to purge alleles. For each individual, we computed a selection index based on the sum of the absolute differences of the individual's AF (which equals 0, 0.5, or 1 for a single SNP) to the CAFs. This was obtained as the sum of the row of the absolute values in the Z matrix. Thus, the selection index weights were the same for all loci. We then fitted this vector in OCS to minimize the average value of the selection index as suggested by Georges et al. (2019).

Comparing the performance of TAFT to the aforementioned selection index (a vector with absolute deviations) may be complicated because the $ZZ^{\prime }$ matrix contains (i) pairwise (off-diagonal) information and (ii) squared deviations, whereas the vector with selection index values does not. Therefore, we introduced a third approach that is in between TAFT and the selection index. The third approach used a selection index in which the AF deviations are squared. This was obtained by squaring all entries in the Z matrix and summing all elements per row. The resulting vector has identical values to the diagonal elements of the $ZZ^{\prime }$ matrix. Thus, only off-diagonal information is missing in the third approach compared to TAFT.

In initial runs, we observed that the unfavorable allele got fixed sometimes, especially when managing the population with either index approach. Since—in practice—breeders would not let the unfavorable variant get fixed, additional constraints were introduced in the OCS problem formulation that aimed at keeping the allele in the population. Since these constraints would favor the selection-index-methods and not the matrix-method (TAFT), and we were interested in how the matrix-method would outperform the index methods, we deemed the restrictions justified. If the allele was to be purged, we set an upper bound for the AF in optiSel of 96%. If intermediate frequencies were to be achieved, we set an upper bound of 96% and a lower bound of 4%. Since optiSel did not allow to specify upper and lower bounds together, only that bound was active which was closer to the AF observed among the selection candidates. The restrictions were realized by calculating the AF of every individual, treating it as a breeding value and fitting the vector in optiSel.

**Table:** 

**OCS problem formulation for the toy example.**
When using TAFT (matrix-method): Minimize $c_{t}^{\prime }ZZ^{\prime }c_{t}$ Or for the selection index strategies: Minimize $c_{t}^{\prime }h$ Subject to $0.5\bar{A_{t}}+0.01\;\geq 0.5{c^{\prime }}_{t}A_{t}c_{t}\;\;\;(\max.\;1\text{\%}\;\Delta F)$ For every SNP: $0.96\geq AF_{t+1}$ If CAF > 0: $0.04\leq AF_{t+1}$ $A_{t}$ is the pedigree relationship matrix in generation *t*. $c_{t}$ is the vector of contributions of animals in generation *t* to the next generation t+1. CAF is the CAF for a particular SNP. $AF_{t+1}$ is the expected AF of a particular SNP in the next generation. h is a vector of selection index values. Here, the sum of deviations from centering allele frequencies or the sum of squares of deviations from centering allele frequencies.

### Application in Friesian horses

#### Traits of interest

The Friesian horse breed suffers from several monogenic conditions causing disorders or lethality (Ducro et al. 2015; Leegwater et al. 2016; Hisey et al. 2020; Steensma et al. 2026). A possible means of reducing disorders and lethality occurrence is to select against the causal variants. In addition to selecting against causal variants, the inbreeding rate should be controlled to avoid a fast loss of genetic variation and to minimize the chance of expression of newly arising disorders. Moreover, breeders select for breeding goal characteristics, i.e. want to make genetic gain. Here, we use the Friesian horse population as a case study to test the performance of the matrix-method and the index-method that considers absolute deviations. Note that this case study is a strong simplification of the real processes going on in Friesian horse breeding.

We here consider 13 genomic regions of interest in the Friesian horse population. Eleven regions harbor unfavorable variants resulting in disease and/or lethality, the twelfth region is a QTL for color intensity responsible for a more intense black coat coloration and the 13th region is the MHC (Table 1). The positions given in Table 1 stem from several studies using different reference genomes. Positions in the Friesian horse may differ slightly but the exact position is not relevant for our simulation study. In this study, the unfavorable allele is coded as 1 and the favorable one as 0. While the frequency of the 1-allele should be moved to 0% for the first 12 loci, the goal in managing the MHC region is to keep or increase diversity, given that high MHC diversity is somewhat associated with better resistance to infectious diseases (Holmes et al. 2019). The goal for the MHC region is to keep as many alleles in the population as possible and to move their frequencies to 50% so that heterozygosity is maximized. Note that 1 of the 13 considered genomic regions, a major locus responsible for insect bite hypersensitivity (IBH), is located within the MHC region. Thus, maintaining diversity in the MHC region is especially complex if the 1-allele at IBH (associated with seasonal allergic dermatitis) has to be purged.

**Table 1.** The 13 genomic regions of interest in the Friesian horse considered in this study.

Trait	Phenotype	Chr:location	Carrier-by-carrier matings avoided?	Allele frequency	Desired allele frequency	Source
Hydrocephalus	Lethal before or at birth if homozygous	1:78 Mb^a^	Yes	0.071^1^	0	Ducro et al. (2015)
Megaesophagus	Diseased if homozygous	3:?Mb (position not identified yet; at 50 Mb in this study)	No	0.37^2^	0	Yousefi (2025)
Recessive lethal 1	Lethal before birth if homozygous	4:71–73 Mb^c^	No	0.074^1^	0	Steensma et al. (2026)
Recessive lethal 2	Lethal before birth if homozygous	5:49–51.5 Mb^c^	No	0.044^1^	0	Steensma et al. (2026)
Recessive lethal 3	Lethal before birth if homozygous	6:53–56 Mb^c^	No	0.13^1^	0	Steensma et al. (2026)
Recessive lethal 4	Lethal before birth if homozygous	9:63–65.6 Mb^c^	No	0.092^1^	0	Steensma et al. (2026)
Distichiasis	Diseased if homozygous	13:0.2 Mb^b^	No	0.33^3^	0	Hisey et al. (2020)
Dwarfism	Lethal or euthanized after birth if homozygous	14:4.5 Mb^a^	Yes	0.067^1^	0	Leegwater et al. (2016)
Recessive lethal 5	Lethal before birth if homozygous	18:18–20 Mb^c^	No	0.064^1^	0	Steensma et al. (2026)
Insect bite hypersensitivity (IBH)	Diseased if homozygous	20:31Mb^a^	No	0.288^4^	0	Schurink et al. (2018)
Recessive lethal 6	Lethal before birth if homozygous	23:9–12 Mb^c^	No	0.091^1^	0	Steensma et al. (2026)
Color intensity	Less intense black coat coloration if homozygous	28:16 Mb^b^	No	0.172^1^	0	Unpublished
MHC		20:29.6–34.6 Mb^b^	-	0.286 (He)^T^	Maximize He	Holmes et al. (2019)

Genome positions are indicated with a range if a single best tagging marker has not been found yet. The range in mega bases (Mb) indicated for MHC is the region containing MHC class I, II, and III (Holmes et al. 2019). All conditions are assumed to be recessive.

^1^Allele frequency based on 70 K SNP data of 8,263 horses analyzed by M.J.S.

^2^Allele frequency based on 70 K SNP data of ∼150 horses analyzed by the University of Kentucky (Yousefi 2025).

^3^Allele frequency based on sequence data of 50 breeding stallions analyzed by M.J.S.

^4^Allele frequency approximately based on Schurink et al. (2018).

^a^Position based on the EquCab2.0 reference genome (Wade et al. 2009).

^b^Position based on the EquCab3.0 reference genome (Kalbfleisch et al. 2018).

^c^Position based on the EquCab_Wur_Friesian reference genome (Steensma et al. 2025a).

^T^This value is observed in the simulation. Heterozygosity (He) in real life may differ. He is calculated as the average expected heterozygosity (2pq) over all loci in the MHC region with a minor allele frequency over 1% which are about 650 loci in our simulation.

In this study, we assume that for each genetic condition a marker has been found that perfectly tags it and that the phenotype is 100% controlled by the allele tagged by the marker. For all the conditions listed with a range in Table 1, this is not necessarily the case in real life. Also, the locus most responsible for IBH only explains 60% of all the phenotypic variation in real life (Schurink et al. 2018). Similarly, the locus associated for megaesophagus does not fully explain the disease in real life (Yousefi 2025). In this study, we assume that all 12 conditions have a fully recessive gene action, i.e. the affected phenotype is only expressed if an animal is homozygous for the unfavorable allele.

Further, genetic tests are currently only available and widely used for hydrocephalus and dwarfism. All breeding mares and sires are currently tested for these two conditions. Breeding with carriers is allowed in the Royal Friesian Horse Studbook (KFPS) policy, but carrier-by-carrier matings are fined (Steensma et al. 2024). A genetic test for distichiasis is also available but horses are currently not tested for this mutation, so we also do not test for distichiasis in our simulations and can therefore not prevent carrier-by-carrier matings. No tests are available for the other conditions yet. In this study, we assume that the genotype state of male selection candidates can be inferred by genotyping at higher density, imputation, or sequencing.

We further simulated withers height as a polygenic trait with a base-generation-heritability of 0.76, a genetic variance of 11.2 (Marije Steensma, personal communication) and an average height of 163.7 cm (Steensma et al. 2025b). Withers height was controlled by 1,000 QTLs in our simulation. It serves as an example for an ordinary polygenic breeding goal trait in our simulation.

#### Simulation of the historical population

To obtain a starting population that somewhat realistically resembles the population history of the Friesian horse breed, we simulated the demographic history of cattle (MacLeod et al. 2013) as given in the example of the function runMacs2() of the AlphaSimR package (Gaynor et al. 2021) (Supplementary Table 1). We assume that the demographic history of horses is somewhat similar to that of cattle. As for cattle, the mutation rate was set to 1.2 × 10^−8^ per base pair as in the example of runMacs2() (https://www.rdocumentation.org/packages/AlphaSimR/versions/2.1.0/topics/runMacs2) (Gaynor et al. 2021). The 31 horse chromosomes were simulated to have the same physical lengths as inferred from the map “map_horse1” provided in the MoBPSmaps package (Pook et al. 2020). That map is based on the study of McCue et al. (2012). Genetic lengths were obtained by assuming a constant conversion rate of 1 Mb per 1 centiMorgan (cM). This is because the total physical length roughly matches the genetic length of ∼2,800 cM (Swinburne et al. 2006).

After running runMacs2() until 10 generations before the start of our breeding program simulation, the population was extracted and read into MoBPS. The last 10 generations of demographic history were simulated in MoBPS to record a pedigree, as MoBPS was used to simulate breeding actions with OCS and deleterious alleles later on. The effective population sizes in the last seven generations were adjusted to approximately resemble those observed in the Friesian breed since the mid-1940s as reported in Figure 3 of Steensma et al. (2024) (Supplementary Table 1). Based on Steensma et al. (2024), we assumed a generation interval of 10 years. Thirty replicates of the population history simulation were run.

#### Management strategies

After population history simulation, SNPs were selected to serve as causal loci as mentioned in Table 1. For this, all SNPs in the region of 3 Mb around the location as stated in Table 1 were considered. That SNP with the AF closest to the AF in Table 1 was selected as the causal locus. The desired AF for all 12 loci was 0%. As in the toy example, we set the CAF to construct Z to 0% to achieve this.

For the MHC region, there are 80 variable SNPs on the genotyping array in the real Friesian horse population. Thus, we sampled 80 random SNPs with a minor AF higher than 1% from the simulated MHC region to serve as SNPs observed when genotyping animals. While the aim to maximize heterozygosity for the MHC region sounds straightforward, the approach is not. Setting the CAF of the genotyped SNPs to 50% would result in maximum heterozygosity only at the genotyped SNPs, but possibly not at the unobserved loci in the region. Gautason et al. (2023) showed that genetic drift can be substantial when using CAF of 50% for the GRM used for genetic diversity management. In preliminary tests, we also observed that heterozygosity at unobserved sites in the MHC region is not necessarily maximized with a CAF of 50% for observed SNPs in the same region (results not shown). Thus, we decided to make “haplotype-alleles” that implicitly considered unobserved sites. For this, we assumed the genotypes were phased. Every unique combination of 80 alleles in the 5 Mb region was considered a separate haplotype. Consequently, multiple haplotypes occurred in the MHC region. Thus, we treated the MHC region as a multi-allelic locus and every haplotype as an allele, hence haplotype-allele. Heterozygosity at a locus is maximized when every allele is at the frequency of (1/number of alleles)100%. The target frequencies for the haplotypes were set to this.

**Fig. 4.**
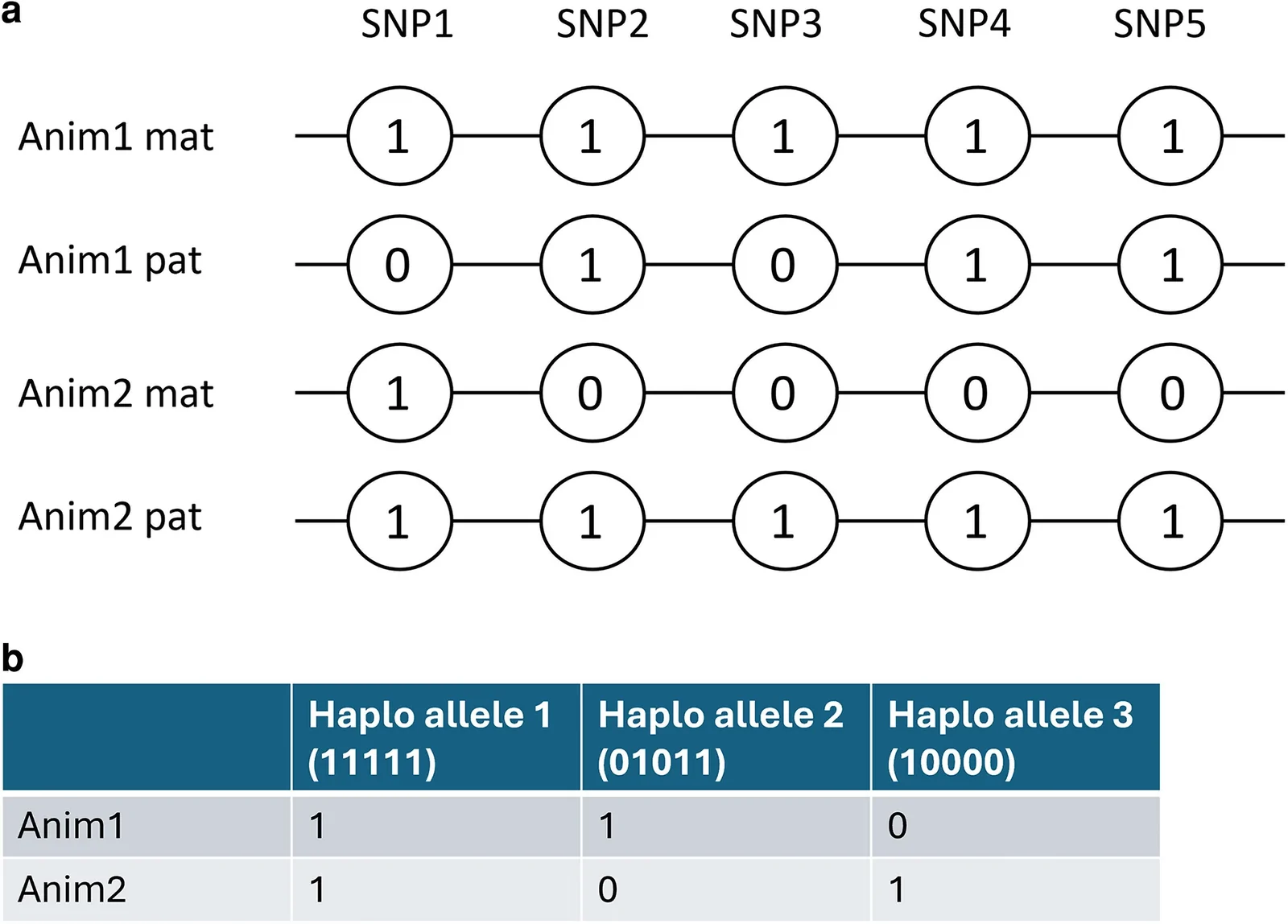
a) Phased genotypes of two animals and b) example for the set-up of a genotype matrix (M) based on haplotype-alleles at the MHC locus. Only five SNPs are genotyped in this example. Panel (a) shows the maternally and paternally inherited haplotype for two animals.

A pseudo-genotype matrix was set up to code the count of each haplotype for each animal. An example is provided in Fig. 4. This matrix is analogous to the ordinary M matrix used when setting up a GRM according to VanRaden (2008). We can similarly form a matrix P that stores twice the desired AF, which in this case is 1/number of haplotypes. Next, Z and $ZZ^{\prime }$ can be calculated as usual. $ZZ^{\prime }$ can then be used for genetic management with the matrix-based method. For management with the index-based method using absolute deviations, the sum of absolute values per row of Z is the index value for MHC for each animal. The desired AF for the haplotypes was determined every generation anew based on the number of observed haplotypes. This is because drift and recombination can lead to the loss or creation of new haplotypes. To avoid giving undue weight to extremely rare haplotypes, only haplotypes with a minimum frequency higher than 0.5% in the population were considered. This was done by removing the respective columns of the M matrix for the haplotypes below this threshold.

The total selection index and the total locus-based matrix are the sum of loci with 0% CAF and the MHC region. When treating the MHC region as a multi-allelic locus as we do with multiple columns in the M matrix, the $ZZ^{\prime }$ matrix for the MHC region would dominate the index since its sum is so large. To avoid this, the $ZZ^{\prime }$ matrix for the MHC region was standardized by dividing by 4 times the number of haplotype-alleles, i.e. columns. The calculation of the total $ZZ^{\prime }$ and the index with absolute deviations are shown below. $Z_{hc}$, $Z_{me}$, $Z_{col}$, $Z_{mhc}$, and so on indicate the Z matrices constructed for the loci associated with hydrocephalus, megaesophagus, color intensity, the MHC region, and so on, respectively. Division by 4 and 2 scales the values so that they express the product of AF deviations of both animals from CAF (matrix-method TAFT) and the absolute AF deviation (index-method).

TAFT (matrix method):


$$\frac{Z_{tot}{Z^{\prime }}_{tot}}{4}=\frac{Z_{hc}{Z^{\prime }}_{hc}}{4}+\frac{Z_{me}{Z^{\prime }}_{me}}{4}+\ldots +\frac{Z_{col}{Z^{\prime }}_{col}}{4}+\frac{Z_{mhc}{Z^{\prime }}_{mhc}}{4\times n_{\mathrm{haplotypes}}}$$

Absolute AF deviation index method:


$$h=\frac{|Z_{tot}|1_{n}}{2}=\frac{\left|Z_{hc}\right|}{2}+\frac{\left|Z_{me}\right|}{2}+\ldots +\frac{\left|Z_{col}\right|}{2}+\frac{\left|Z_{mhc}\right|}{2\times n_{\mathrm{haplotypes}}}$$

$1_{n}$ is a vector of 1 s of length *n* with *n* being the number of loci (columns) in $Z_{tot}$. Vertical bars indicate that absolute values are used. $|Z_{tot}|1_{n}$ thus expresses summation of the absolute values per row in matrix $Z_{tot}$.

#### Simulation of the Friesian horse breeding program

We considered discrete generations in our simulation for simplicity. The generation interval in Friesian horses is about 10 years on both male and female side (Steensma et al. 2024). About 3,500 foals are born every year which makes 35,000 foals in a 10-year time period. Mares have a foal every 2 to 3 years which makes 3 to 4 foals per mare per generation. Thus, we selected 10,606 mares (10 years/3 years-between-foaling = 3.3 foals per mare per generation; 35,000 horses per generation/3.3 foals per mare per generation = 10,606 mares per generation). Mares were selected based on their phenotype for withers height. In this study, we assume that animals are favored with higher withers. We acknowledge that only selecting for withers height is a simplification of reality as withers height is not the only selection criterion used by breeders and that increasingly withers height indefinitely is also not desired.

In contrast to mare selection, stallions were selected with OCS. We limited the number of stallion selection candidates to avoid selecting a large number of stallions. This may happen by the use of OCS but is unrealistic in horse breeding. About 90 stallions are active every year in the real Frisian horse population (Steensma et al. 2024). Therefore, we assumed that about 150 would be active in a 10-year time period. We preselected the phenotypically best 150 stallions for withers height per generation and additionally the 3 tallest sons of every grand-sire. This may be seen as an effort to not let a paternal lineage die out due to shortness. This also results in a preselected group larger than 150 and an average phenotype (withers height) that is lower than that of the 150 best ones which may be selected in practice. To achieve the same rate of genetic gain that would theoretically be realized by truncation selection of the tallest 150 stallions, we restricted the solution space of the OCS problem so that the average weighted phenotype of the stallions selected with OCS had to be equal to or higher than the average phenotype of the 150 tallest ones.

Further, we restricted the pedigree inbreeding rate in OCS to 0.25% per generation. Also, according to future plans of the studbook, we limited the maximum number of matings a sire is allowed to have to 120 per year (Steensma et al. 2024). This means only 3.4% (120/3500) of all foals per year may have this stallion as father. This corresponds to a restriction of a stallion's contribution to a maximum of 1.7% (half of 3.4%, because all male contributions should sum to 50% in OCS). No restriction was set on the minimum contribution per stallion, such that stallions of the preselected group could still get a zero contribution, i.e. are not selected. With these restrictions in place, contributions were optimized so that AF was changed either with the matrix-based method or the index method using absolute deviations.

**Table:** 

**OCS problem formulation for the Friesian horse breeding program**
When selecting with the matrix-method: Minimize $c_{t}^{\prime }ZZ^{\prime }c_{t}$ For the index-based strategy: Minimize ${c^{\prime }}_{t}h$ Subject to $\;0.5A_{pf,pf,t}+(1-0.5A_{pf,pf,t})0.0025\;\geq 0.5{c^{\prime }}_{t}A_{t}c_{t}\;\;(\max.\;0.25\text{\%}\;\Delta F)$ $0.5({\bar{w}}_{150\;best}+w_{pf})\leq {c^{\prime }}_{t}w$ $0.017\geq c_{t,max}\;\;\;(\mathrm{except}\;\mathrm{for}\;\mathrm{the}\;\mathrm{pseudo}-\mathrm{female})$ $A_{t}$ is the pedigree relationship matrix for all preselected stallions and the pseudo-female in generation *t*. $A_{pf,pf,t}$ is the diagonal element of the pedigree relationship matrix related to the self-relationship of the pseudo-female (average relationship among 500 animals) generation *t*. $c_{t}$ is the vector of contributions of preselected stallions and the pseudo-female in generation *t* to the next generation t+1. The contribution of the pseudo-female is fixed at 0.5. h is a vector of selection index values. Here, the sum of deviations from centering allele frequencies. w is a vector containing the phenotype for withers height for every preselected stallion and the pseudo-female.

So far, mares are only genotyped for hydrocephalus and dwarfism but not at any of the other positions. In addition, the studbook has no control over which females are selected as mares. Thus, the relationships of each stallion to each mare and the carrier status of each mare are unknown when selection decisions and contributions are to be assigned to stallions. In addition, solving the OCS problem with 10,606 females is very time intensive, if at all possible. Steensma et al. (2024) solved this by using the average relationship of a stallion to the population. This is similar to the pseudo-female approach used for OCS by Gautason et al. (2023). The pseudo-female approach is appropriate when all females have approximately equal contributions. It works by condensing the information of all females into one value that represent them all (the pseudo-female). In a relationship matrix, the columns and rows of all females are removed and instead just one column and row for the pseudo-female is used. The off-diagonal entry belonging to the pseudo-female is the average relationship of the sire in question to all females. The diagonal element belonging to the pseudo female is the self-relationship, i.e. the average relationship among all females. Since this works for relationship matrices, we also used the pseudo-female approach for our $ZZ^{\prime }$ matrix and the Z matrix. The contribution of the pseudo-female was set to 50% in optiSel (Wellmann 2019).

Still, to condense the value of all females in a single average value for a pseudo-female would strictly require knowledge of all female values. In this study, we sampled just 500 random animals from the same generation (male and female) and assumed they represented all females. That is, we assumed that these 500 animals were genotyped at all 13 chromosome regions of interest (Table 1).

**Fig. 5.**
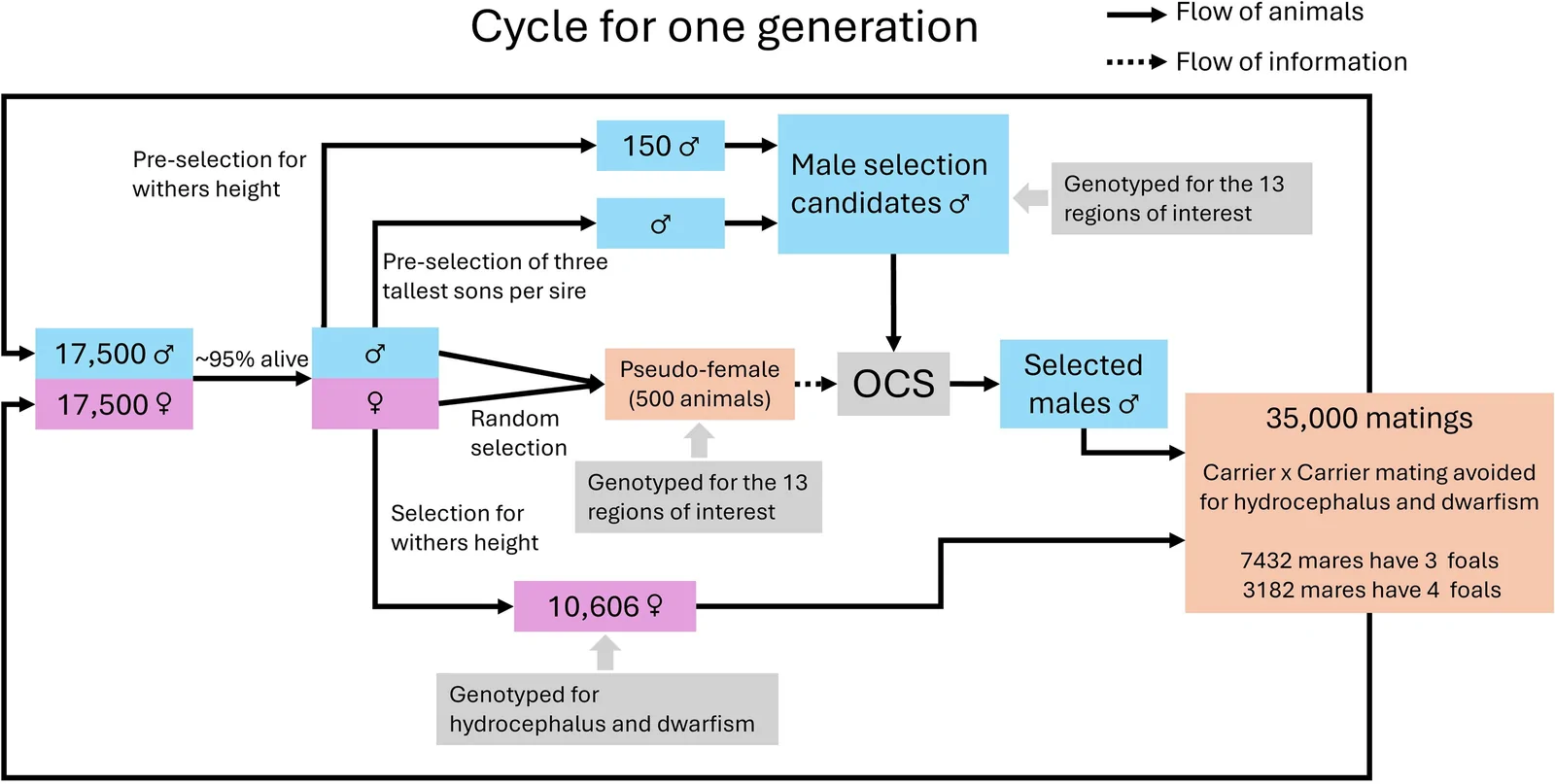
Schematic visualization of actions happening in one generation of the simulated Friesian horse breeding program.

Matings were completely random in the simulation, except that carrier-by-carrier matings for hydrocephalus and dwarfism were prevented to mimic current practice. Animals that were homozygous for one (or more) of the recessive lethals died in our simulation which can result in a smaller population size than 35,000. One generation of the simulated Friesian horse breeding program is shown schematically in Fig. 5. All 30 replicates of population history were used as a starting point for all selection methods, i.e. every selection method was run with 30 replicates.

## Results

### Toy example

#### Purging alleles at 5 loci

**Fig. 6.**
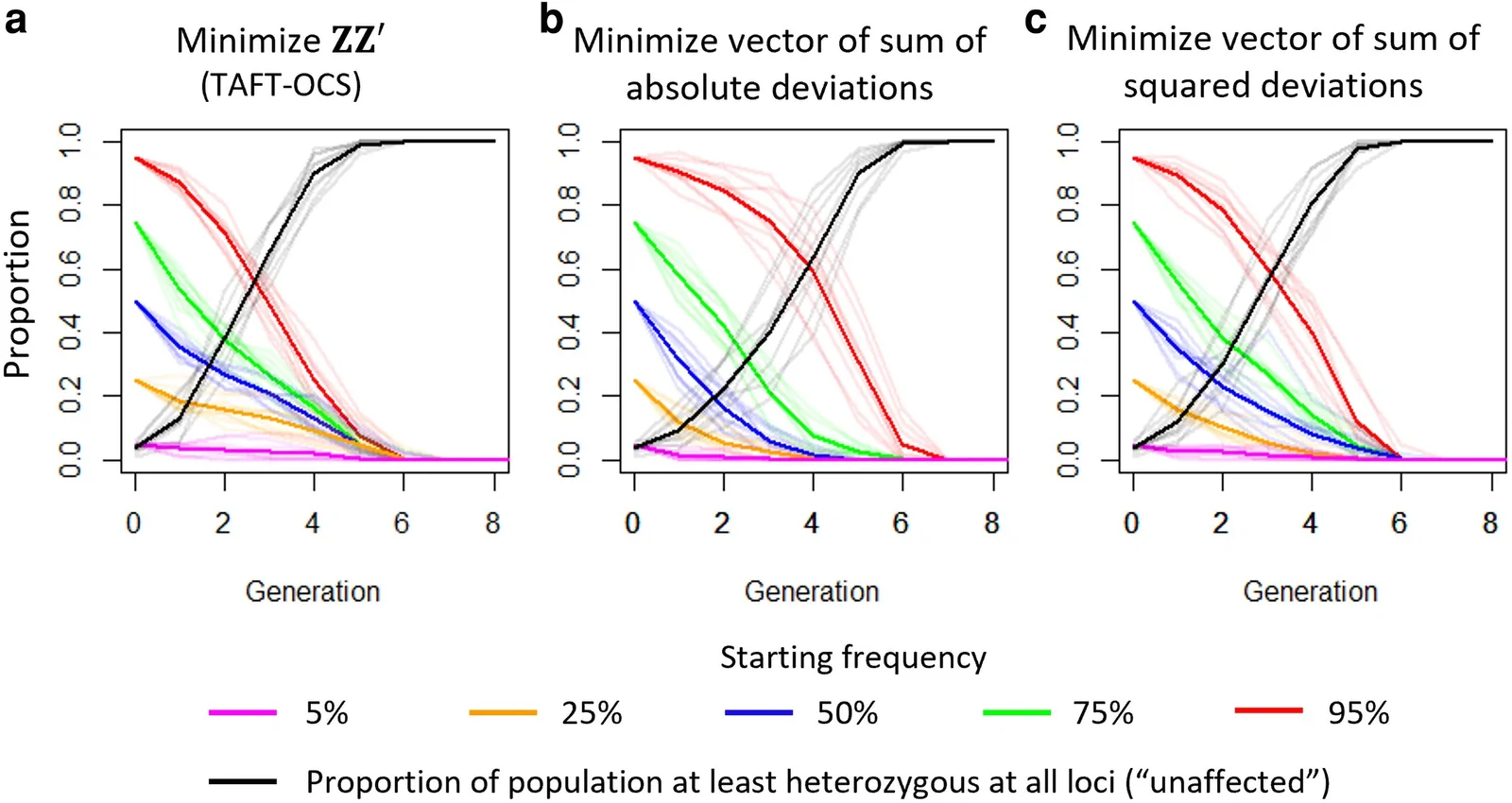
AF trajectories over generations when the aim is to purge all alleles. Alleles have initial frequencies of 95%, 75%, 50%, 25%, and 5% (colored lines). Black lines indicate the fraction of the population that is not homozygous for the unwanted allele at any of the 5 loci. Individual replicates are shown in faded lines, and means for all 10 replicates are shown with dark lines. (a) A population managed so that contributions minimize $ZZ^{\prime }$, (b) a population managed so that the vector with the sum of absolute deviations is minimized, and (c) a population managed so that the vector with the sum of squared deviations is minimized. Inbreeding is restricted to show the same increase in all scenarios.

All 3 selection strategies managed to purge all unwanted alleles at the 5 loci from the population within 6 to 7 generations (Fig. 6). However, the AF trajectories differed across the selection strategies. TAFT-OCS resulted in purging of all alleles at approximately the same generation, regardless of the starting frequency. Using an index of AFs, however, resulted in alleles being purged one after the other starting with the allele with the lowest starting frequency. Finally, using an index with squared deviations showed a pattern that is in between the other strategies.

**Fig. 7.**
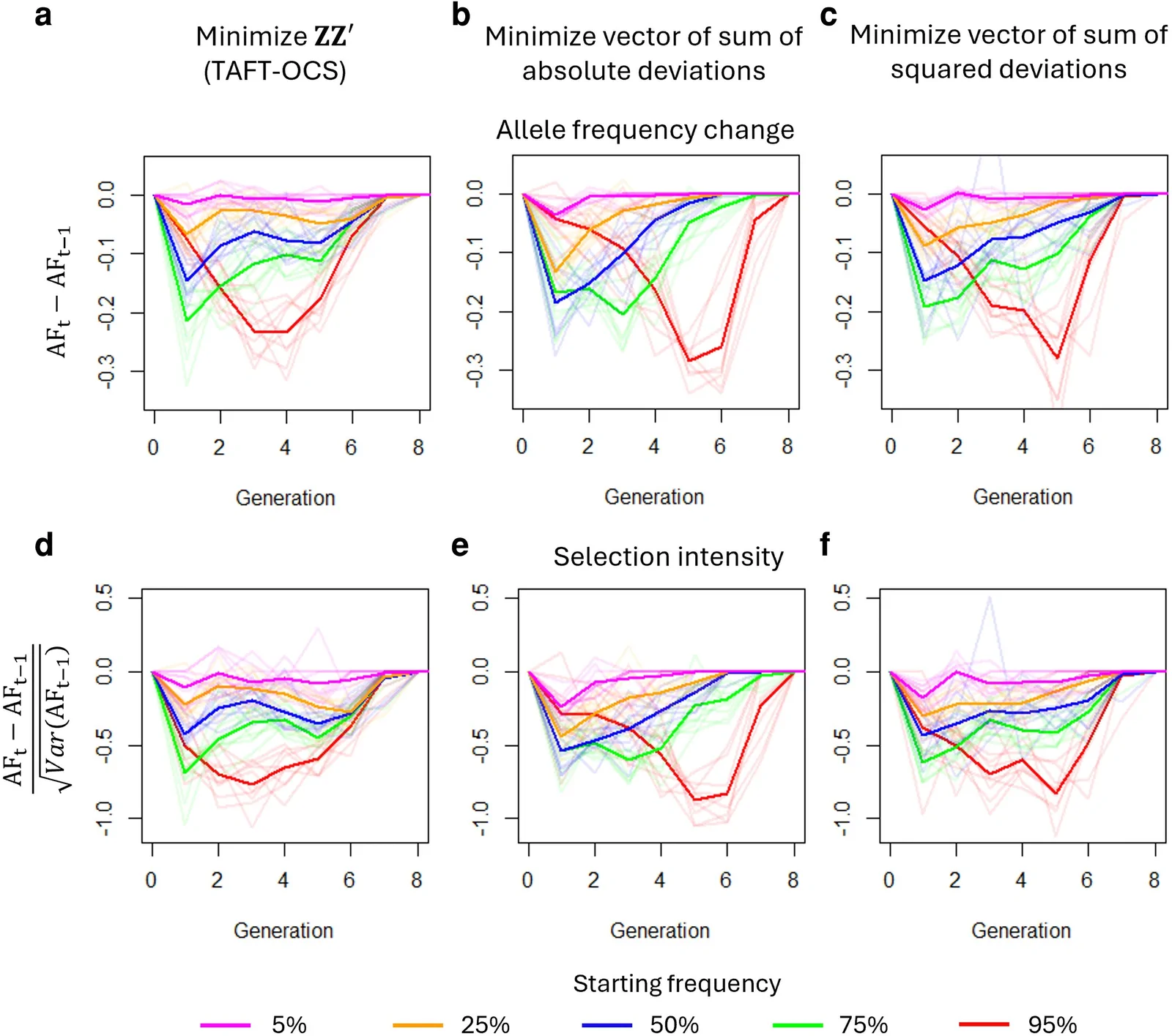
(a to c) AF change $\left(AF_{t}-AF_{t-1}\right)$ over generations when the aim is to purge alleles, for 5 alleles with initial frequencies of 95%, 75%, 50%, 25%, and 5%. d to f) Selection intensity calculated as the between-generation difference of allele frequencies divided by the standard deviation of allele frequencies observed among individuals. Individual replicates are shown in faded lines, and means for all 10 replicates are shown with dark lines. (a) and (d) refer to a population managed so that contributions minimize $ZZ^{\prime }$, (b) and (e) refer to a population managed so that the average of the vector with the sum of absolute deviations is minimized, and (c) and (f) refer to a population managed so that the average of the vector with the sum of squared deviations is minimized.

The difference between the matrix-method (TAFT) and the index-method of absolute deviations becomes more obvious when visualizing the change of allele frequencies and the selection intensity realized at each locus (Fig. 7). Minimizing the absolute deviation resulted in AF changes that appear to depend on how “intermediate” the AF is, i.e. more change the closer the AF to 50%. How intermediate the AF is also seemed to affect the change per locus when using the matrix-method but also the difference to the CAF (here 0%) influenced how much change is made. This resulted in comparatively uniform selection intensities over generations achieved by TAFT compared to those observed with the index-methods (Fig. 7) (selection intensities are negative because lower values are favored). The best example for this is the allele with a starting frequency of 95%. The index-methods change the frequency of this allele relatively little in the first generations. Once the other alleles are close to 0%, the frequency of the 95%-allele is changed rapidly. This is the reason why the proportion of animals that are not homozygous for the unwanted allele increases less steeply than if the population were managed with TAFT (compare black lines in panel a and b in Fig. 6). Assuming “homozygous for the unwanted allele at least at one locus” can be understood as “affected,” the populations managed with the index-methods show a higher proportion of affected animals in earlier generations. Note that an animal needs to be homozygous at only one of the 5 loci to count as affected and the high frequency of the 95%-allele is likely the reason for this higher proportion.

#### Moving allele frequencies of 5 loci to the same target frequency

**Fig. 8.**
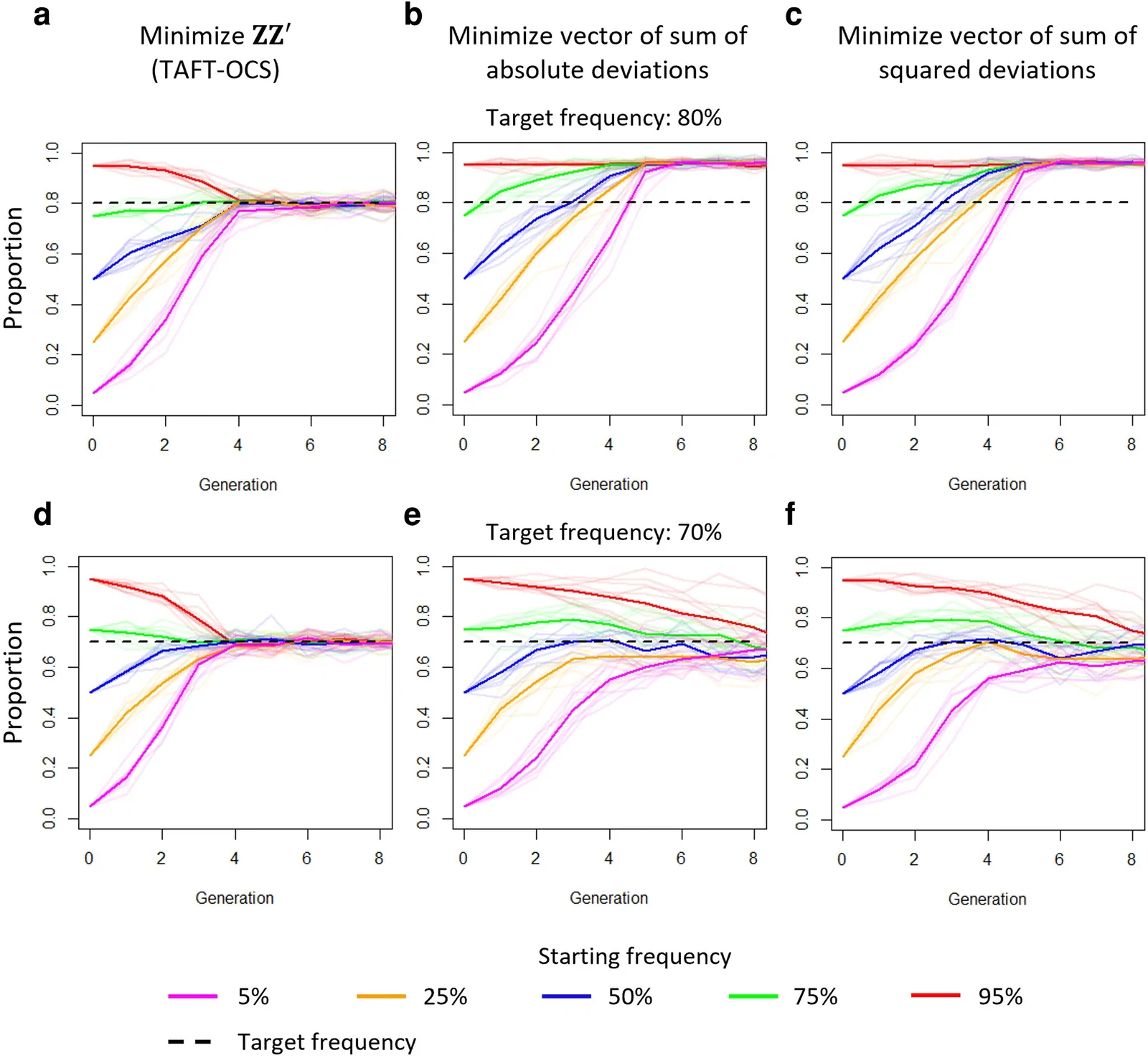
AF trajectories over generations when the aim is to shift and then keep all allele frequencies at 80% (a to c) or 70% (d to f). Alleles have initial frequencies of 95%, 75%, 50%, 25%, and 5% (colored lines). Black dashed lines indicate the desired frequency (80% or 70%). Individual replicates are shown in faded lines, and means for all 10 replicates are shown with dark lines.

Though likely of less practical relevance, the next experiments in which we tried to keep alleles at specific frequencies between 0 and 100% provide useful insights to understand how the methods work. TAFT successfully brought all five alleles to the desired 80% within four generations whereas the two index-based methods failed (Fig. 8). Figure 8 suggests that the AF remains at around 95% in later generations when the population is managed with index-based methods. Actually, the AF is at around 96% as this was the upper limit we set in the OCS problem formulation. Without these constraints, the frequencies would have gone up to 100%. Following this observation, we set the desired AF to 90%, 78%, 75%, 70%, and 60% and only observed non-failing behavior for the target frequency of 70% or lower (Fig. 8). Non-failing needs to be understood here as the indication that a (close to) desired situation may be achieved in the future. Solutions of OCS were valid for all replicates in all tested scenarios.

#### Moving allele frequencies of 5 loci to locus-specific target frequencies

**Fig. 9.**
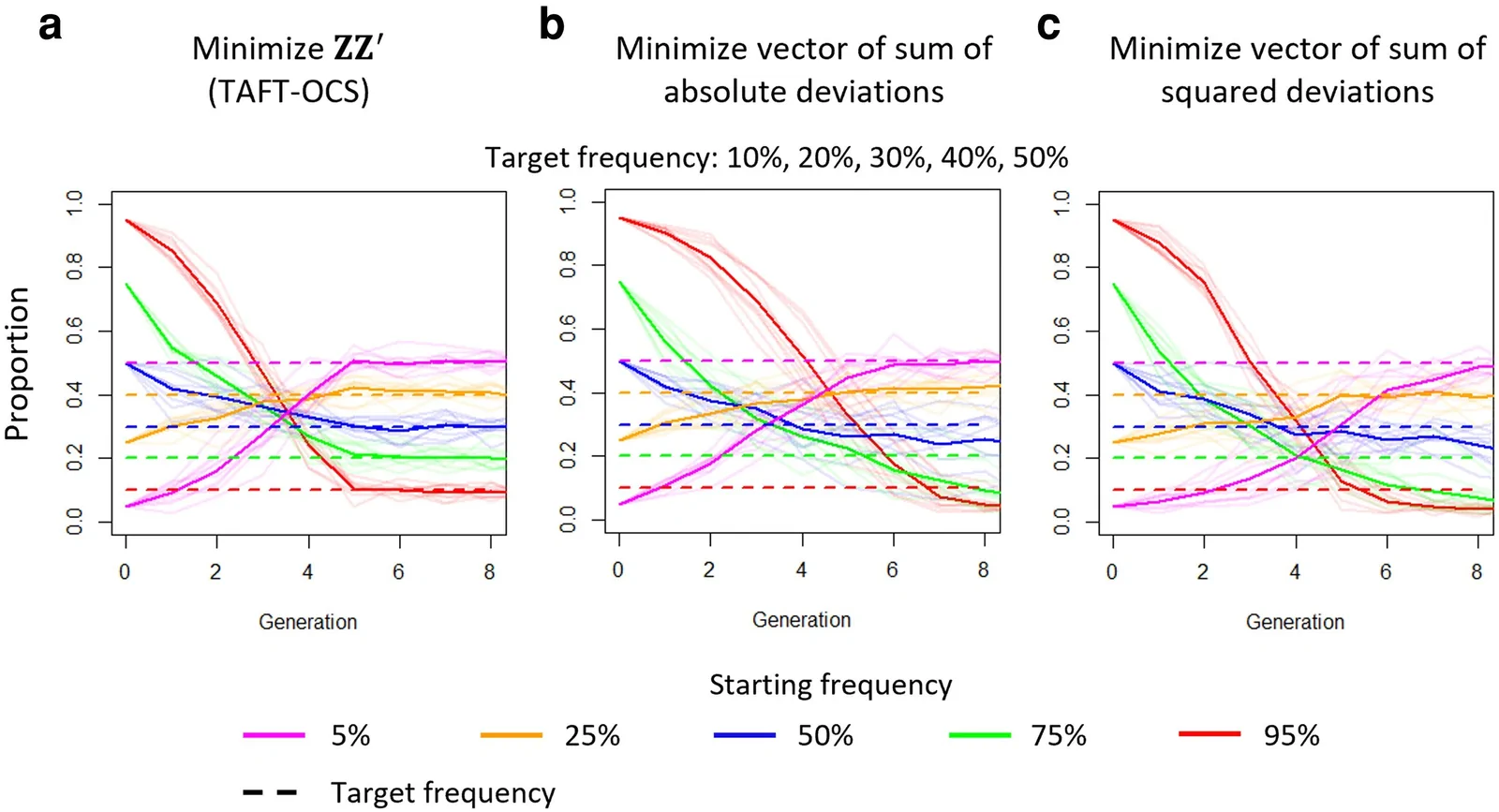
AF trajectories over generations when the aim is to shift and then keep all allele frequencies at 10%, 20%, 30%, 40%, and 50% when selection with (a) TAFT-OCS, (b) an index of absolute deviations, or (c) an index of squared deviations. Alleles have initial frequencies of 95%, 75%, 50%, 25%, and 5% (colored lines). Dashed lines indicate the desired frequencies (10%, 20%, 30%, 40%, and 50%). Individual replicates are shown in faded lines, and means for all 10 replicates are shown with dark lines.

When setting the target allele frequencies to 10%, 20%, 30%, 40%, and 50% for the five alleles, the matrix method (TAFT) successfully shifted the AF to the desired AF after five generations (Fig. 9). Index-methods did apparently not fail completely but performed poorer than the matrix method in three aspects. First, they achieved the desired frequencies later. Second, the alleles with desired frequencies of 20% and 10% (green and red lines) showed continuously decreasing frequency up until the lower bound (4%) was reached. Lastly, index-based management showed much more fluctuation of individual replicates between generations (compare the spread of faded lines in Figs. 6, 8, and 9).

#### Purging alleles at many loci

**Fig. 10.**
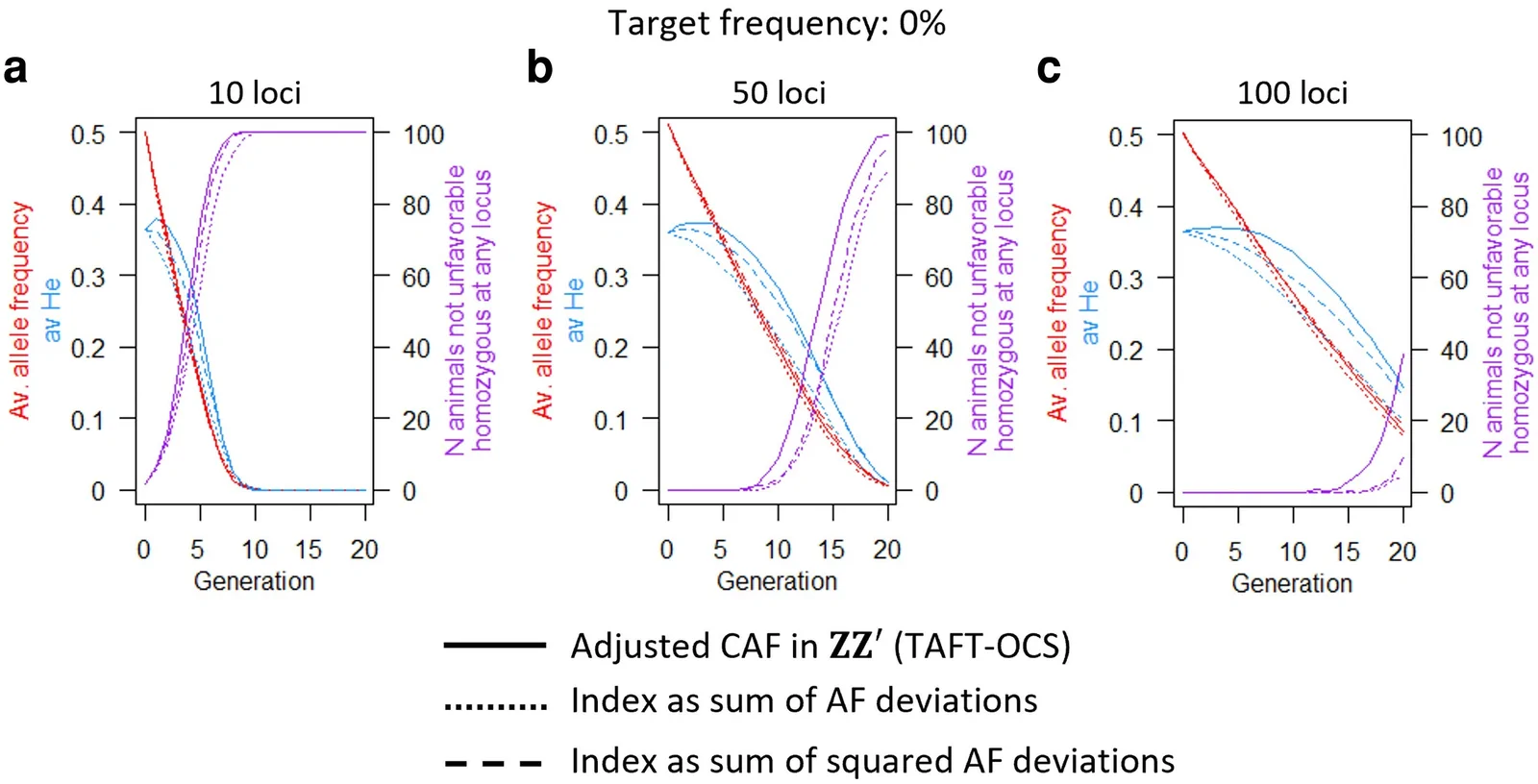
Allele frequency (red), expected heterozygosity (2pq) (blue), and the number of “unaffected” animals (purple) that are not homozygous for the unfavorable variant at any locus. Panels (a), (b), and (c) show scenarios in which 10, 50, and 100 random SNPs were selected for purging the 1-allele from the population. Lines indicating AF and expected heterozygosity are averages over all these 10/50/100 loci. All lines are averages over the 10 replicates.

When more loci are to be purged, the rate of AF change per locus decreased and it took longer until animals that were not “affected” emerged (Fig. 10). While the change of AF was approximately identical among all methods, the matrix-based method (TAFT) resulted in a faster increase of unaffected animals. Solutions of OCS were valid in all replicates. The desired allele never got lost from the population even when considering 100 loci.

The average expected heterozygosity (He) measured at loci at which AF are to be changed was highest for TAFT followed by the squared-index method. Interestingly, the index-method with absolute deviations resulted in a strict monotonic decline of He whereas the squared-index method and the matrix-method even increased He slightly in early generations. This aligns with observations made before in Fig. 6 that when aiming to purge alleles, the matrix-method moves alleles with AF above 50% quickly to 50%, and the AF of alleles with low AF is not changed as rapidly, that is, in comparison to the index-method. Thus, the matrix method (i) moves AF faster to 50% and (ii) keeps sites segregating for longer. Both result in a comparatively higher He.

### Application in the Friesian horse population

**Fig. 11.**
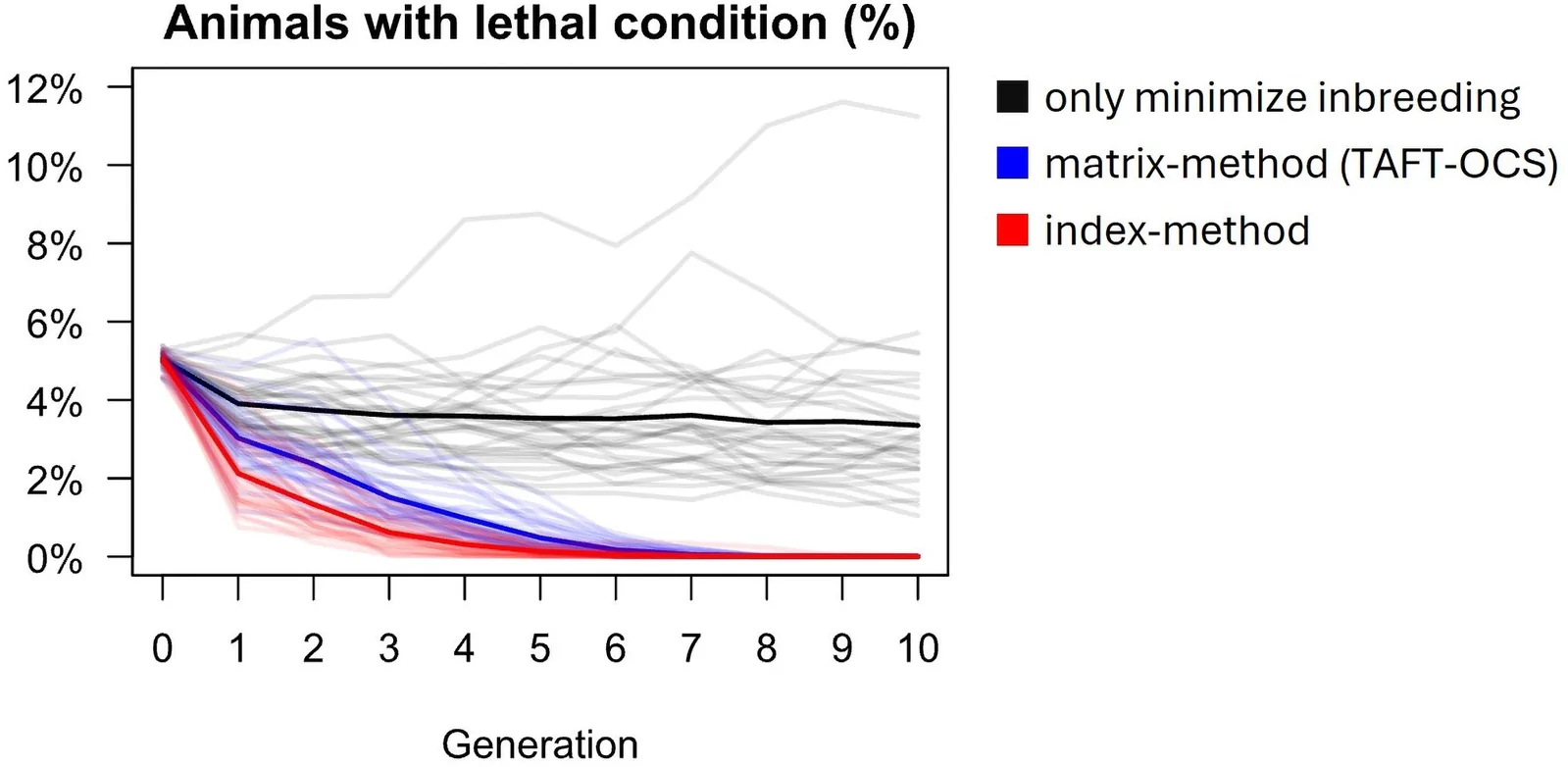
Percentage of Friesian horses that are homozygous lethal for at least 1 of the 8 loci that have lethal alleles $\left(\frac{n_{\mathrm{dead}\;\mathrm{or}\;\mathrm{unborn}}}{35,000}\times 100\text{\%}\right)$. Individual replicates are shown in faded lines, and means for all 30 replicates are shown with dark lines.

The index-method reduced the occurrence of homozygous lethal animals the fastest from about 5% to about 0.5% after just 3 generations (Fig. 11). The TAFT-OCS method was comparatively slower but showed very similar behavior. In another scenario, in which the objective was to minimize inbreeding as much as possible while setting the lower bound restriction for withers height and without intentional selection for specific loci, lethality dropped too, namely, from 5 to 3.3% after 10 generations. This is because of natural selection, i.e. homozygous lethal animals die or are euthanized and do not contribute to the next generation. Lethality increased markedly in one replicate when the aim is to minimize inbreeding but this was purely due to chance (Fig. 11).

**Fig. 12.**
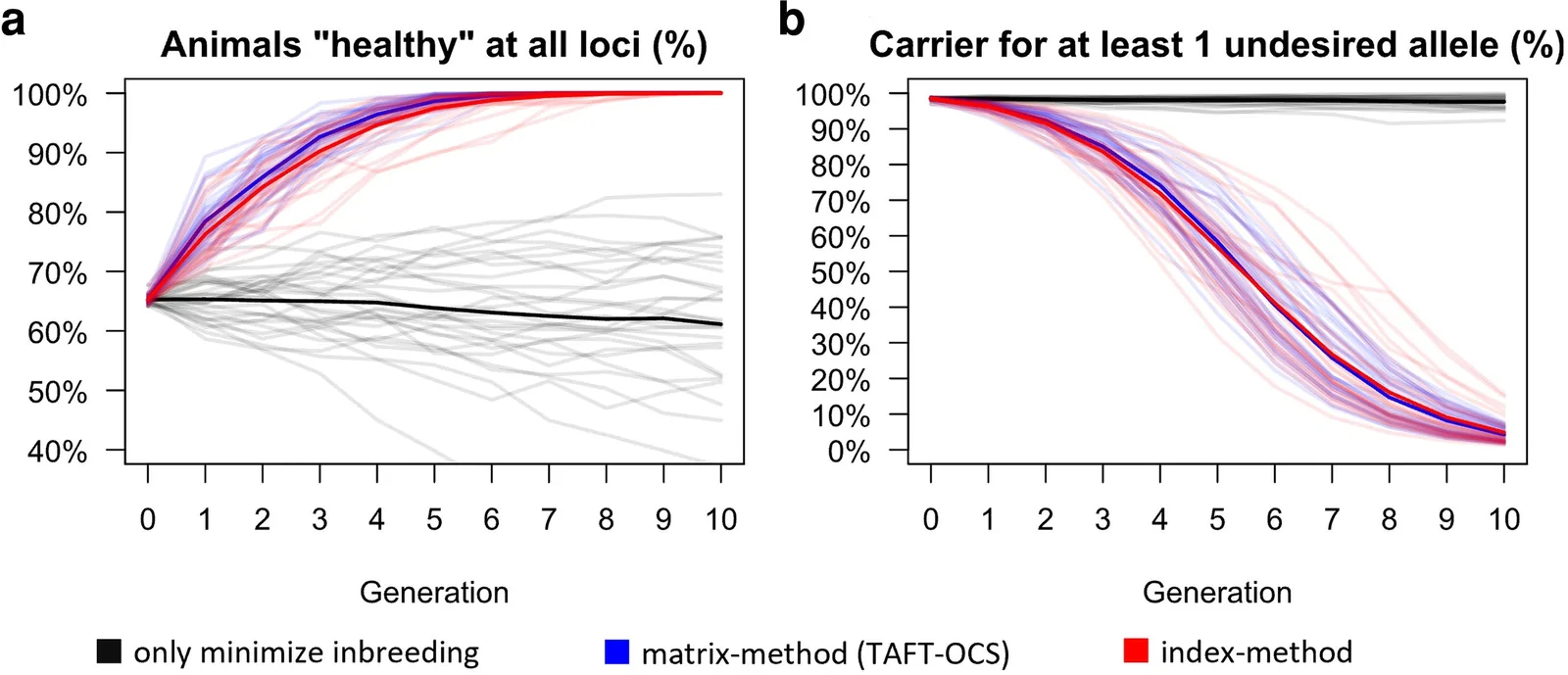
(a) Percentage of horses that are at least heterozygous, or homozygous for the beneficial allele, at all loci. If treating color-intensity as genetic condition, this is the percentage of animals that is born (not homozygous lethal) and healthy from all pregnancies $\left(\frac{n_{\mathrm{healthy}}}{35,000}\times 100\text{\%}\right)$. (b) Percentage of animals that are carriers of the undesired variant for at least one locus. Individual replicates are shown in faded lines, and means for all 30 replicates are shown with dark lines.

In contrast to lethality, the percentage of healthy animals (non-homozygous for the undesired allele at any of the 12 loci including color intensity) increased fastest with the matrix-method (Fig. 12) though not much faster than with the index-method. Almost the entire population could be considered genetically healthy after ∼6 generations of selection. Carriers remained much longer in the population. For example, even after 6 generations of selection, about 30% of all animals still carried an undesired allele at least at one locus. This frequency was reduced to below 5% after 10 generations.

**Fig. 13.**
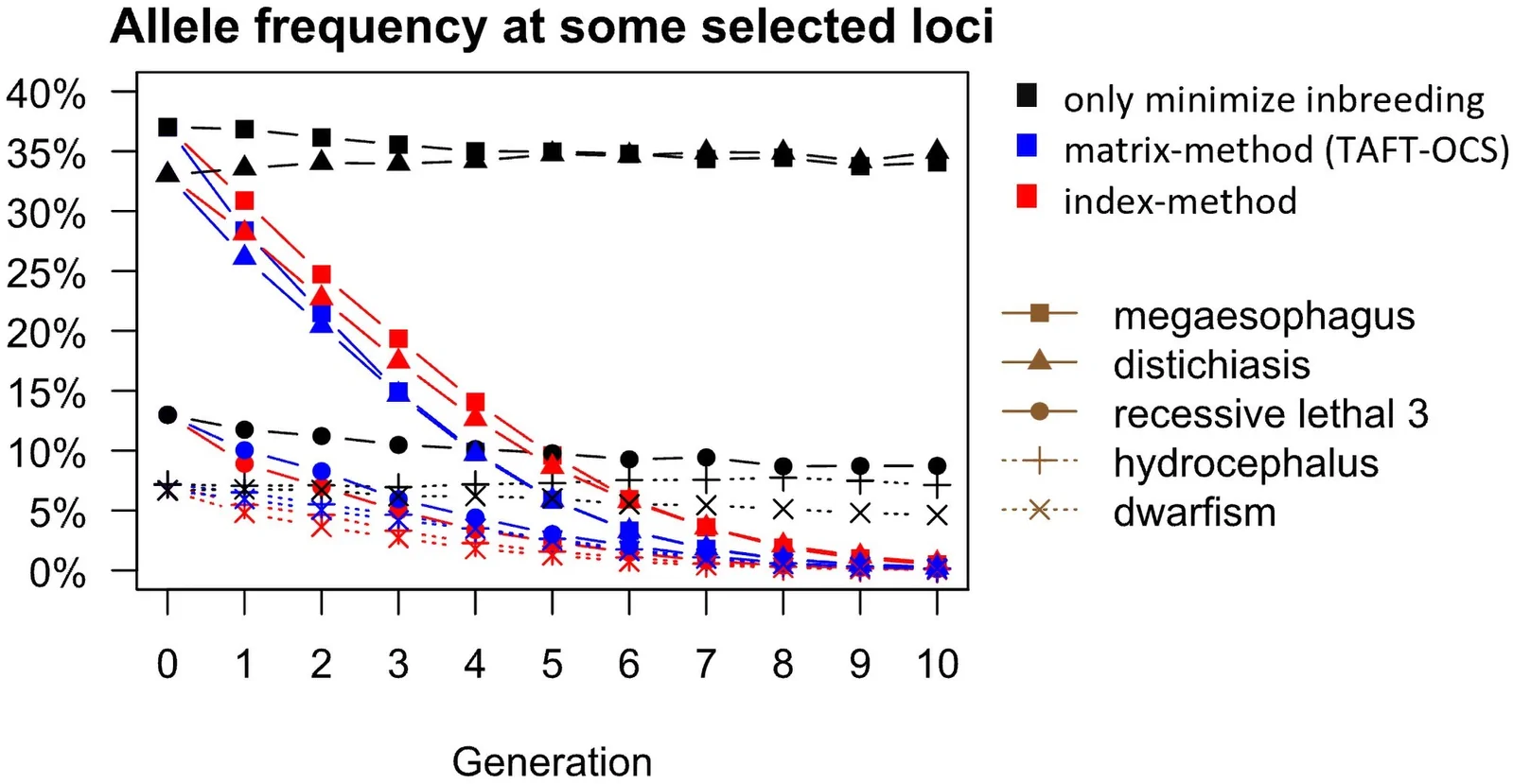
AF at the locus associated with megaesophagus, distichiasis, recessive lethal 3, hydrocephalus, and dwarfism. Averages over the 30 replicates are shown. The two loci for which genetic tests exists and for which carrier-by-carrier matings are avoided (hydrocephalus and dwarfism) are indicated with dotted lines.

The frequencies of the 12 unwanted alleles were reduced quickly by both the index-method as well as the matrix-method. All undesired alleles were either purged or had allele frequencies well below 1% after 10 generations of selection. For better readability, only results of 5 selected loci are shown (Fig. 13). Allele frequencies remained somewhat stable when the only objective was to reduce the inbreeding rate, except for lethal alleles for which carrier-by-carrier matings were not avoided (recessive lethal 1 to 6). The frequencies of these decreased because of natural selection, which is in concordance with the reduction of overall lethality in the population (Fig. 11). Frequencies for hydrocephalus and dwarfism, although lethal, did not decrease by natural selection since carrier-by-carrier mating avoidance ensured that these lethal alleles never occurred in homozygous form.

Compared to the index-method, the matrix-method reduced the frequency of highly frequent alleles (megaesophagus and distichiasis) faster. Thus, frequencies of common alleles (hydrocephalus, dwarfism, and recessive lethal 3 in Fig. 13) decreased more slowly with the matrix-method than with the index-method. This in turn explains the slightly lower decrease of lethality observed with the matrix-method in Fig. 11: the low frequent alleles, which are changed less strongly with the matrix-method, happen to be lethal.

**Fig. 14.**
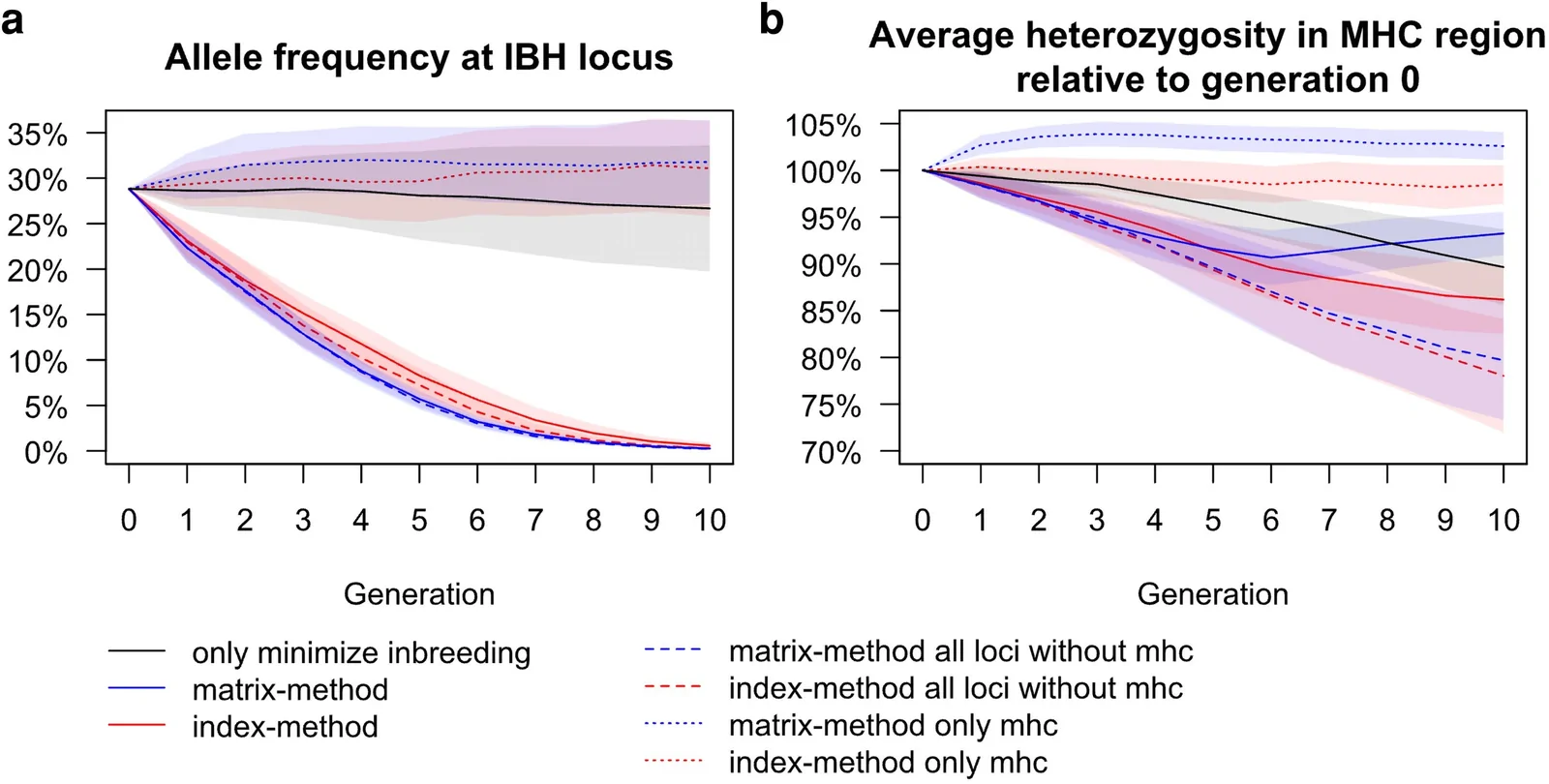
(a) AF of the allele associated with IBH in the Friesian horse simulation. (b) Average expected heterozygosity (2pq) of all genotyped and ungenotyped loci in the 5 Mb MHC region expressed relative to the heterozygosity observed in generation 0. Lines indicate average of 30 replicates and the transparent regions indicate the 95% confidence interval for the mean (mean ± 1.96·se).

To better understand processes at the IBH locus that is located within the MHC region, we ran two additional scenarios. In the first scenario, all 12 loci were included in the selection objective but the MHC region was excluded. In the second scenario, the sole objective was to increase diversity at the MHC region. When the MHC region was excluded from the objective, the AF of IBH reduced slightly faster than in the standard objective that includes the MHC region and all other 12 regions (Fig. 14). Diversity in the MHC region decreased continuously for the index-method and until generation 5 for the matrix-method after which it partly recovered when MHC was included in the selection objective.

Relative to the average heterozygosity at the MHC region in generation 0, the matrix-method even managed to increase diversity by about 4% when the sole objective was to focus on MHC. The index-method barely achieved keeping the initial diversity (Fig. 14). The diversity measure was calculated as the average expected heterozygosity of all approximately 650 loci (80 used for genotyping, the rest unobserved) in the MHC region. Expected heterozygosity was based on the observed AF and calculated under the assumption of Hardy–Weinberg equilibrium (2pq). We expressed diversity relative to the observation in generation 0 because the value of 0.286 given in Table 1 is just the average across replicates. Individual replicates showed higher or lower diversity in generation 0. This initial variation makes comparisons more difficult. Expressing diversity loss within each replicate removes this initial variation $\left(\frac{avHe_{t}}{avHe_{t=0}}\right)$. For reference, 105%, 100%, and 80% correspond to an average heterozygosity of 0.3, 0.29, and 0.23, respectively.

**Fig. 15.**
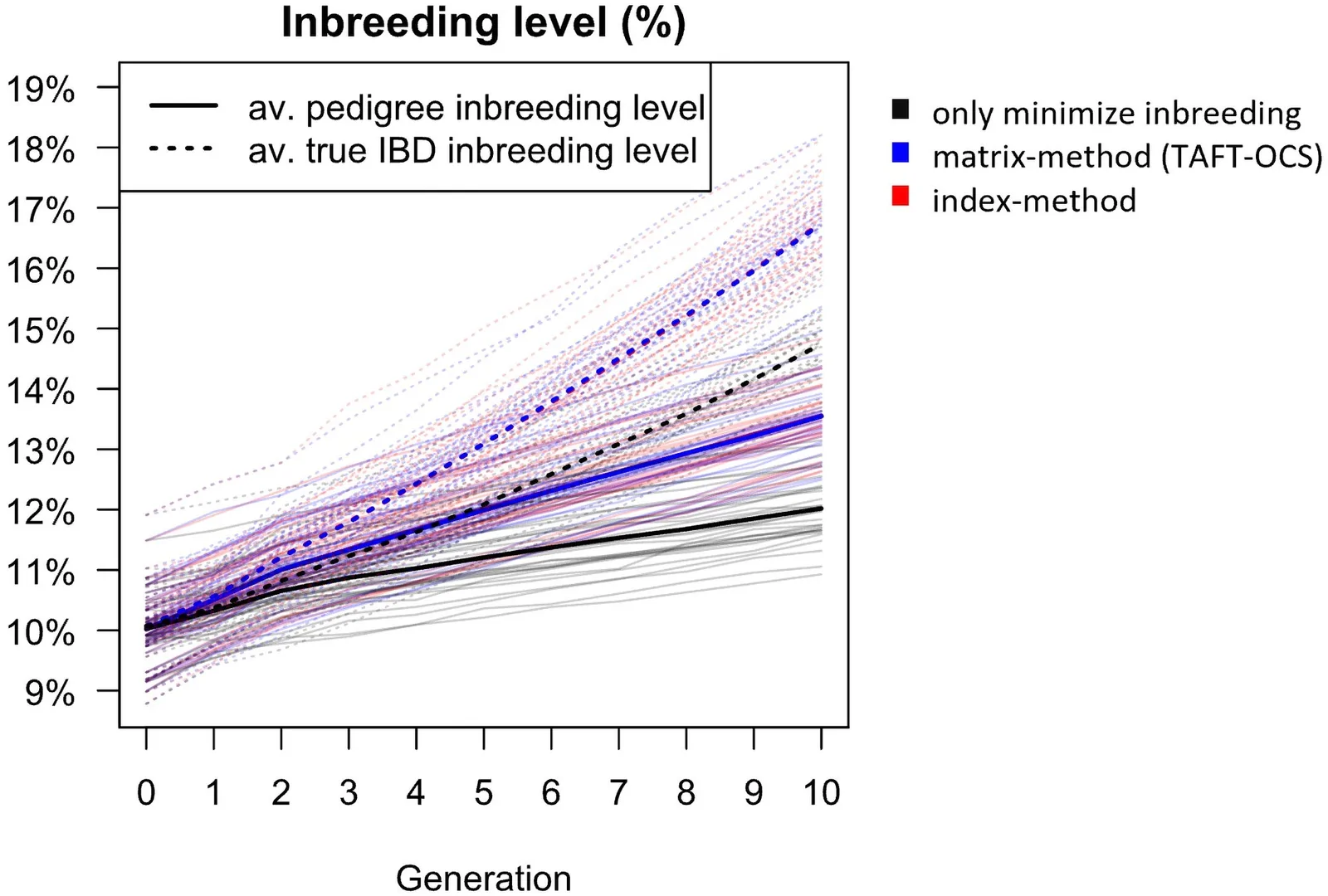
Average inbreeding level over time in the Friesian horse breeding program simulation. Individual replicates are shown in faded lines, and means for all 30 replicates are shown with dark lines. The red index-method lines are barely visible because they are behind matrix-method lines.

The average pedigree inbreeding level, as the average of the diagonal elements of the relationship matrix, increased from about 10 to 12.0% when the sole objective was to minimize inbreeding (Fig. 15). The average pedigree inbreeding level increased to 13.5% in generation 10 for the two methods targeting allele frequencies. The higher inbreeding level is expected as, for those methods, inbreeding was just set as a restriction but was not subject to minimization. Figure 15 also shows that the true identity-by-descent (IBD) inbreeding rate was higher than the pedigree inbreeding. The true IBD inbreeding rates were 0.53%, 0.77%, and 0.77% per generation for the 3 strategies, respectively. The pedigree inbreeding rates were 0.22%, 0.40%, and 0.40%. Inbreeding rates are calculated as $\Delta F=1-{\left(\frac{1-F_{t}}{1-F_{0}}\right)}^{1/t}$. The true IBD inbreeding level is calculated by MoBPS (Pook et al. 2020) with function inbreeding.emp() which traces chromosome segments back to the founders. In our simulation, founders are the animals in the generation in which pedigree recording started (Supplementary Table 1). This was 10 generations before generation “0” of the breeding program.

Withers height increased from 163.7 to 200.2 cm after 10 generations of selection (Table 2) with little variation between methods. This is an increase of about 3.7 cm (3.7 mm) per generation (year) which is a change of about 1.11 (0.111) genetic standard deviations. This is the result of our simulation and it should be noted that the KFPS does not have the goal of breeding 2 meters tall Friesians. In real life, more traits other than withers height are considered and breeding goals may be adjusted after some shift in trait values has been achieved.

**Table 2.** Average withers height over time.

	Average withers height (cm). Standard error of the mean is given in parenthesis				
	Generation 0	Generation 1	Generation 3	Generation 6	Generation 10
Minimize inbreeding only	163.7 (0.29)	168.2 (0.29)	175.9 (0.30)	186.8 (0.33)	200.2 (0.40)
Matrix-method (TAFT-OCS)	163.7 (0.29)	168.3 (0.29)	176.0 (0.30)	186.8 (0.35)	200.2 (0.41)
Index-method	163.7 (0.29)	168.3 (0.29)	176.0 (0.31)	186.9 (0.33)	200.4 (0.38)

## Discussion

Here, we confirm simulation results from previous studies on populations managed with optimal contributions (OCS) using a GRM. Specifically, that when the GRM used is calculated according to method 1 of VanRaden (2008), then selection on low average relationship drives the allele frequencies of the managed loci to approach the “centering frequencies” used to center those genotypes (Gautason et al. 2023). We posit that this behavior is due to the genomic relationships (calculated according to VanRaden's method) being proportional to the sum of the products of deviations from the centering frequencies. In particular, the sum of squares of individuals’ AF deviations is proportional to individuals’ self-relationships. Thus, selection for low average values of such GRMs, e.g. as with OCS or based on mean kinship, favors selection of such combination of individuals whose allele frequencies are as close to the centering frequencies as possible. We call this approach “targeted allele frequency tuning (TAFT)”. The focus with TAFT-selection shifts from the animal to the alleles. Through two toy examples and a Friesian horse breeding simulation, we explored how TAFT can be used in combination with OCS to modify the allele frequencies in a population toward desired target values (TAFT-OCS).

Our study adds to existing literature (Meuwissen et al. 2020; Gautason et al. 2023) showing that OCS decisions are not invariant to the choice of centering, or base, AF. This is contrary to what we understand from the statement made by Colleau et al. (2017) regarding no effect of the choice of AF used for centering genotype counts on the optimal contributions. Our study shows that when the VR1 matrix is compared to other relationship measures, the centering frequencies need to be reported and considered. Using VR1 with centering frequencies as observed in the sample is not ideal for diversity management. For completeness, it should be mentioned that while the choice of CAF does affect ranking and relative differences of relationships, it does not affect the ranking of EBV of animals (Strandén and Christensen 2011). This is because genomic evaluations estimate the effect of the deviation from the CAF when having one more copy of the allele. This detail is usually not important because whatever the CAF, the accuracy, as the correlation between true and estimated BV, stays the same. Or better, the correlation of EBVs from two genomic evaluation runs (each with individually estimated variance components) with different CAF is expected to be 1. Only when unbiased EBVs are desired or when genomic EBVs should be comparable to EBVs from pedigree evaluations do allele counts have to be centered first before multiplication with estimated SNP effects $\left(\mathrm{EB}V_{\mathrm{ind}}=\sum {\hat{\alpha }}_{i}(2AF_{i,\mathrm{ind}}-2\mathrm{CA}F_{i})\right)$. The point is that genomic evaluations already work with the deviation of frequencies, but the choice of CAF does not really matter there as it does for genomic diversity management (e.g. in genomic relationship matrices for use in diversity management).

### Similarities between TAFT-OCS and selection index

The toy examples showed that purging with TAFT-OCS (a matrix-method) or index-methods resulted in comparable reduction of average AF. However, this was achieved with different underlying strategies. An index consisting of absolute deviations from desired AF initially changed alleles with AF around 50% the most. The method using a matrix with desired AF used for centering initially put more emphasis on high AF, i.e. more deviating from the desired AF, alleles than on low AF alleles. Ultimately, this means that if, hypothetically, the outcomes from selection with TAFT were translated into selection index weights, more weight, and thus more selection pressure, is placed on high AF alleles. This would not happen with an economic selection index when the change of one unit, e.g. 1 percentage point, is equally valuable for a locus with a 10% AF as for a locus with a 90% AF (assuming additive gene action). It is theoretically possible to alter selection index weights in such a way that the AF trajectories observed with an absolute-deviation-index with said altered weights would closely resemble those observed when selection is carried out with the TAFT method. Or better, since the weights would change every generation, it is theoretically possible to obtain the same trajectories observed with the TAFT method with a sequence of absolute-deviation-indices given the particular population at hand. To conclude, TAFT can also be seen as a selection index that puts unequal weights on the loci, putting higher weights on loci with current AFs further away from their target AFs.

Interestingly, although for a single locus, Fernández et al. (2006) developed an approach similar to ours for obtaining contributions so that predefined allele frequencies are met. We want to highlight a statement of their discussion, namely, that their index, and thus also our TAFT method, may be seen as a quadratic form of a desired gains index.

The analogy of TAFT as a selection index that adjusts weights automatically ends if the target AF is not 0% or 100%. This was shown with Figs. 8 and 9 in which target frequencies were values between 0 and 100%. The analogy of the economic selection index with unequal weights may only be continued if index weights are adjusted every generation—a requirement necessary when traits have optimum values (see e.g. Wellmann (2023) and Werner et al. (2024)). It should be noted that what we call the absolute-deviation-index-method in this manuscript is not identical to commonly known economic or desired gain indices when the target AF is between 0 and 100%, i.e. when selection is done for optimum traits. The values of individuals with the absolute-deviation-index and the squared-deviation-index may be seen as measures of “similarity” to the desired value. The absolute-deviation-index adjusted the observation, i.e. the allele dosage, to absolute deviations whereas this is not done in typical indices in which the weights are adjusted to keep the population mean at an optimum value. Thus, as mentioned before, a more sophisticated comparison to TAFT may be a desired gains index with adjustments of weights every generation. This would allow to change the direction of genetic progress of the population. Cases of failure, as shown in Fig. 8 when the target frequency is 80%, could then be avoided.

Working major effect loci into a preexisting index may require less change to preexisting breeding procedures in practice than using TAFT especially when generation intervals are long. Yet, TAFT has the advantage that weights according to frequency are automatically assigned and that fluctuations around an optimum are reduced.

### Failure of index-methods

Our index methods failed to maintain intermediate allele frequencies because they lack information about relationships between individuals (i.e. off-diagonal matrix elements). Consider the following example with fully inbred individuals like double haploids and a target of 80% AF for all loci. To achieve the AF of 80%, 20 individuals that are homozygous for the 0-allele and 80 individuals that are homozygous for the 1-allele need to be selected. An individual homozygous for 0 has an absolute deviation from 80% of 0.8 (|0–0.8|). An individual homozygous for 1 has an absolute deviation of 0.2 (|1–0.8|). Thus, the index value of the individual with the AF closer to the CAF is lower. Since contributions are optimized such that the average index value is minimized, index-based methods would only select individuals being homozygous for 1. This is the case regardless of whether the absolute or squared deviation is used in the index as squaring the deviation only affects the weight given to each locus.

The heterozygous individuals are the reasons why index-based methods are apparently not failing when the target frequency is 70% (see Fig. 8). The index value of heterozygotes is 0.2 (|0.5–0.7|) whereas that of homozygous 0 and 1 individuals is higher, namely, 0.7 and 0.3, respectively. This explains why AF does not go up to 100% when the CAF is 70% and management is based on index-based methods. However, so far this explanation would yield one to expect observed AF to be around 50% which would be failure and not, as here, around 70% (panels e and f in Fig. 8). If the sole selection criterion was the index, then we would indeed expect to see stabilization of AF at 50% because only 1 male and 1 female individual would be selected that are each heterozygous at all 5 loci. These individuals do exist although they are rare. Assuming all loci to have an AF of 50%, only 3% ((2 × 0.5 × 0.5)^5^) of all individuals show the heterozygous genotype at all 5 loci. With a population of 100, this would have resulted in 3 ideal individuals. Selecting only 3 individuals would have resulted in a higher inbreeding rate than the 1% set as the restriction. Thus, to meet the inbreeding restriction, additional individuals were selected which then did not have the maximally lowest index values. These additionally selected individuals are more likely homozygous for 1 than for 0 since that results in the comparatively lower index value. These additionally selected individuals are the reason for the apparently non-failing behavior. For reference, between 40 and 90 animals were selected in each generation.

We expect that the 70% target scenario as well as any target other than 0%, 50%, or 100% would result in failure with the index-based methods. That is however only if the population consisted of enough heterozygous individuals so that the inbreeding restriction could be met when only heterozygotes are selected, i.e. failing is expected with no or a more relaxed inbreeding restriction, or for a numerically large population. We would also expect failure if the population consisted only of fully homozygous individuals like inbred lines or double haploids as in some plant breeding settings at the target frequency differs from 0% or 100%. Likewise, selection indices can only yield satisfactory solutions in autotetraploid species, like potatoes, if target frequencies are 0%, 25%, 50%, 75%, or 100%. Whether enough heterozygous individuals, or no heterozygous individuals are present at all, and regardless of ploidy, the TAFT-OCS would have achieved the desired AF as explained with help of Fig. 3.

To test whether the differences observed between the squared-deviation index and the matrix-method are due to the optimization method of optiSel, we also internally tested minimizing for a matrix whose diagonal elements are the elements of the squared deviation index and whose off-diagonal elements are uninformative by setting them to 0, hereafter named matrix H. Our findings are described in the following. Setting off-diagonal elements to 0 resulted in slower AF changes and virtually all individuals being selected. This is because the weighted matrix average $c^{\prime }Hc$ that is optimized for in OCS is lower the more animals are selected since this means averaging over more 0 elements. To avoid this issue but still making the off-diagonal elements uninformative, we set the off-diagonals in H to the average of the two respective diagonal elements. Using such a matrix in $c^{\prime }Hc$ gave contributions that were identical up to the fourth decimal digit to solutions of the index-method for which optimization is done with respect to $c^{\prime }h$ (with h being the vector of values for the squared-deviation index values). This is because $c^{\prime }Hc$ expresses a contribution-weighted average and $c^{\prime }h$ expresses the same average. The only tested solver that did not result in the same solutions for both methods was cccp2. Interestingly, the optimal contributions obtained with cccp2 when setting off-diagonal elements to 0 were the same as those when setting off-diagonal elements to the average of the respective diagonal elements. This is incorrect and in disagreement with solutions obtained with the other solvers which is why we ignored solver cccp2 in this study. Future users developing new applications for OCS are advised to pay attention to sensitivity of results to the solving techniques.

### Future development

Though we focused on purging, these methods can also be used to achieve allele fixation. Whenever we purged an allele, we fix the alternative (for biallelic loci). A possible scenario may be the introgression of alleles from external sources. This could be combined with advanced OCS (Wellmann et al. 2012; Wang, Bennewitz, et al. 2017; Wang, Segelke, et al. 2017; Kohl et al. 2020; Cao et al. 2024), i.e. OCS where the aim is to introduce the variant from the external population but also to minimize the contribution of the donor breed to the population at hand to maintain breed integrity. Considering introgression also raises the question of how to “cut out” the variant of interest from the genetic background of the donor population. The most straightforward method would be to introduce the allele via genome editing, but such an approach is not always desired or possible due to economical, regulatory, and ethical considerations. Thus, breeders often have to rely on cross-overs. To promote animals with a donor-elite-crossover close to the position of the locus, one could include the presence of a crossover in the selection objective. Alternatively, the donor genome percentage in the region around the position of the locus could be considered in the selection objective.

As shown with the example of the MHC locus in which multiple haplotype-alleles per locus were considered, TAFT can also be extended to multi-allelic cases. With the additional restrictions on frequency to avoid accidentally losing alleles, this might make TAFT-OCS an interesting method to select toward achieving “ultimate genotypes,” i.e. individuals that are fixed for all beneficial haplotypes or alleles (Hayes et al. 2024; Villiers et al. 2024; Yadav et al. 2025). The restrictions on allele frequencies could also be used to achieve a particular target frequency directly in the next generation which may make application and communication to stakeholders easier. For example, if the AF is low, say at 3%, and the beneficial allele is usually found in less performant individuals as would be the case for an introgressed resistance allele, a breeding company may decide that it should be at 20% in the next generation. To achieve this, simply a lower-bound restriction of 20% needs to be added to the OCS problem formulation. This might be easier than increasing the selection index weight for this allele. From a diversity management perspective, in addition to restricting the rate of inbreeding which influences how much allele frequencies are allowed to change from the centering frequencies as shown in this study, imposing upper and lower bound restrictions on AF opens the door for new strategies. For example, restrictions could be imposed that none of the say 50 k genotyped SNPs is allowed to be fixed or lost since they may harbor useful genetic effects for the future. Avoiding the loss of genetic variants might be the ultimate goal for long-term conservation of populations. The commonly used restriction of the rate of increase of coancestry only approximates the avoidance of loss.

Several studies have investigated the optimal AF trajectory to maximize long-term genetic gain (Dekkers and van Arendonk 1998; Sánchez et al. 2006; Goddard 2009). They show that an ordinary selection index does not lead to the highest long-term gains. The highest long-term gains are achieved when the index weights depend on the frequency of the allele such that beneficial alleles with low frequencies receive more weight (Goddard 2009). Sánchez et al. (2006) showed algebraically that the average squared selection intensity across generations should be minimized in order to minimize the loss of response to selection at all other polygenic loci. Dekkers and van Arendonk (1998) in fact observed that the selection intensity at a major QTL was constant over time when selecting according to their method for maximizing response to selection for a quantitative trait over multiple generations. Our TAFT method combined with OCS resulted in relatively constant selection intensities at the loci compared to the index-methods (Fig. 7d to f). When considering large numbers of loci in selection, a higher average heterozygosity at the loci was achieved with the matrix-method than with the index-methods (Fig. 10). In this case, alleles with AF above 50% are prioritized by the matrix-method because those loci were further from the target frequency (0%). Our findings suggest that TAFT-OCS may fit the requirements set in aforementioned studies and may consequently be more suitable than a selection index for sustaining variation and thus long-term gain.

Further research could focus on fitting all loci with BLUP estimated SNP effects in the matrix and using TAFT to drive genetic progress. We speculate that the weight by which to multiply the matrix for each SNP may be the absolute estimated SNP effect (for $ZZ^{\prime }$) or the square root of it (for Z). Centering allele frequencies only have to be adjusted for the sign of the SNP effect, i.e. CAF would be either 0% or 100%. Such an approach would be of interest for higher-long-term gains. To connect this idea to selection on breeding values, consider that a breeding value can also be thought of as a selection index in which the allele count is multiplied with the estimated SNP effect. With the exception of the weighing factor, this is identical to the tested absolute-deviation index. Future research should pay special attention to handling effects of linked SNPs that all capture part of the effect of a QTL.

We would like to highlight an apparent contradiction of selection objectives and diversity management objectives. Directional selection aims to increase the sum of selection differentials at all loci $\left(\sum \Delta AF_{i}\alpha_{i}\right)$, i.e. aims to change allele frequencies. Diversity management, in form of limiting the inbreeding rate, aims at limiting the summed squared AF changes from CAFs $\left(\sum \Delta AF_{i}^{2}\right)$, i.e. aims at not changing allele frequencies. At first, it may seem that the two objectives are mutually exclusive. However, they differ in that the square is in the latter and the consideration of the allele substitution effect size is only in the former. Considering both objectives jointly, as in OCS, might lead to more even frequency changes among SNPs, particularly when the trait is polygenic. This is due to squaring of the frequency changes which strongly “punishes” large changes. Thus, it can be hypothesized that restricting the inbreeding rate when selecting on polygenic traits might result in AF trajectories that are close to the optimal AF trajectory pointed out in the aforementioned studies, but this requires more formal evaluation.

### Real-life practicability

Results from the Friesian horse breeding program simulation demonstrate that genetic selection is a powerful tool to reduce the occurrence of lethal recessive mutations and genetic disorders. Genetic tests for all disorders are currently not available, so carrier-by-carrier matings cannot be avoided for all loci. Of course, reducing the AF and avoiding carrier-by-carrier matings would be optimal as affected animals are avoided and the size of the breeding population is not reduced (Upperman et al. 2019). Only avoiding carrier matings helps reduce the occurrence of the disease but does not help reduce the AF. Thus, the complementarity of sire and dam must always be considered. In case the carrier status of females is unavailable or in case labor costs to consider complementarity are too high, like in large dairy cattle operations (Cole et al. 2025), the mating can be considered random. Consequently, reducing the AF in the population is the only option to also reduce the disease frequency.

The results of TAFT-OCS are more similar to those of the index-method in the Friesian horse simulation than in the toy example. This may be because the main aim in the Friesian population was purging for which we also found similar outcomes between the methods in the toy example. Another reason may be that the solution space of OCS is constrained so much with the restriction of the inbreeding rate and genetic gain that only small room is left for differences in changing allele frequencies.

This study used withers heights as an example of a quantitative breeding goal trait. In real life, many more traits are important for selection decisions. For real-life application in Friesian horse breeding, a general selection index comprising all considered traits like movement, exterior, rideability, and character should be used instead of withers height. In this study, withers height was simply used as a placeholder of the index to demonstrate that increasing breeding goal traits while simultaneously restricting the inbreeding rate and changing allele frequencies of deleterious variants with TAFT can be achieved jointly.

The 13 genomic regions of interest were mostly located on separate chromosomes and were thus not linked. An exception was the MHC region, which is in the same region of a QTL associated with IBH. Due to this linkage, selection on IBH is thus expected to affect the MHC region, and vice versa. We show that TAFT can manage diversity in this region while simultaneously purging the deleterious QTL. Purging the unwanted variant at IBH just takes a bit longer when diversity in the linked MHC region is considered (Fig. 14a). When placing all loci with unwanted alleles on one chromosome to create linkage between them in the settings of the toy example, we observed the same AF trajectories as reported here with the only difference that it took more generations to achieve the desired frequencies (not shown). This implies that whenever genomic regions underlying desirable traits are known, they can be readily integrated into TAFT even if they are linked.

We assumed that the 13 genomic regions of interest had no pleiotropic effects or genetic correlations on other traits. This reflects the knowledge about these regions to this date. If such effects are known, these should be included in the valuation of the alleles. An allele with very strong pleiotropic adverse effects of equal importance may then not be worth changing. If a locus just shows some correlation to other traits of importance, a good solution would be placing more weight on those traits in the selection index, or setting lower (or upper) bound restrictions on trait averages in OCS to avoid correlated selection responses. Applying TAFT on alleles of which not all pleiotropic effects are known is not riskier with regard to unwanted side effects than e.g. culling carriers. Reevaluating the effect of alleles after some genetic progress has been made is always recommended.

**Fig. 16.**
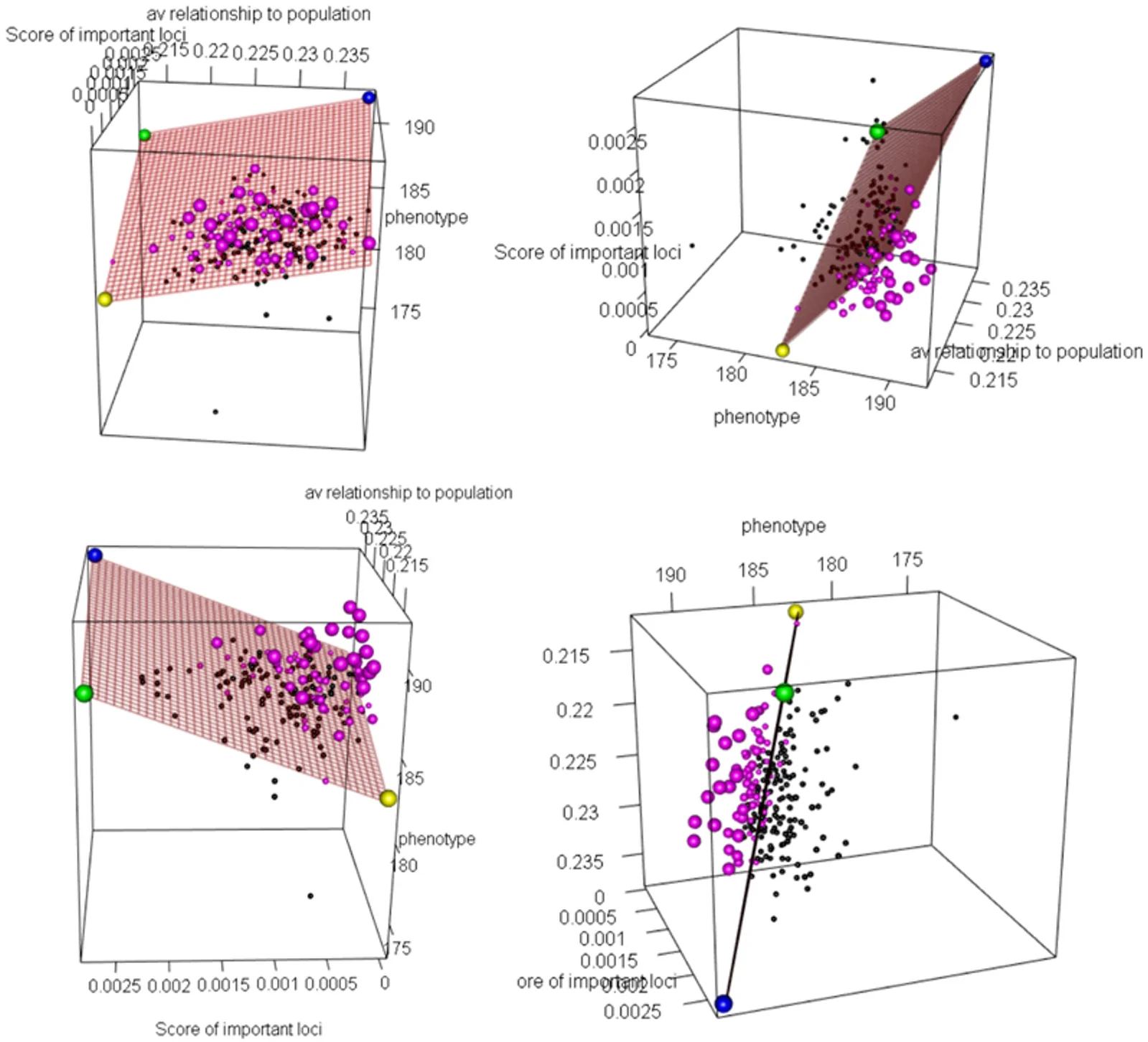
Three dimensional plot showing all considered stallions by OCS (balls). Pink balls are stallions that got a non-zero contribution. The size of balls corresponds to the contribution a stallion got assigned. Several stallions got the maximum allowed contribution corresponding to 120 matings per year. Shown are the dimensions (1) average relationship to the population, (2) the phenotype, and (3) the score of important loci (here: average element of the row of the $ZZ^{\prime }$ matrix pertaining to the stallion and the animals used as pseudo-female). Values shown in this plot are taken from a random replicate in generation 4 of the breeding program. The yellow, green, and blue balls are added to aid understanding of the different rotations. The same data points are used in each rotation.

Perhaps particularly relevant to horse breeding is that breeding goals vary among breeders depending on the traits they wish to improve in their mare. Hence, it may be useful to apply TAFT only for managing deleterious allele frequencies at specific targeted loci. Then, the TAFT-derived selection value (see also Fig. 16) might serve as an extra information tool for health-related characteristics and can be used jointly with values for breeding goal traits chosen according to individual breeder preferences.

### Inbreeding rate and management

We found a higher rate of true genomic IBD inbreeding than pedigree inbreeding (Fig. 15). This is also observed in e.g. dairy cattle (Cole 2024) and simulations by Sonesson et al. (2012). A possible explanation for the elevated true inbreeding rate may be that withers height was under selection. That means that chromosomal regions around genes influencing withers height may become more frequent or less frequent than what one would expect based on pedigree relationships. This is supported by the fact that both the average IBD and the pedigree inbreeding levels were at 10% after the burn-in period in which no selection was applied. In other words, the higher genomic inbreeding rate can be attributed to faster change of regions flanking a QTL than expected in neutral regions (Sánchez et al. 2006; Roughsedge et al. 2008). The higher genomic inbreeding rate may be controlled by switching to a GRM.

The pseudo-female approach utilized in this study would work for a GRM as well. In this study, we sampled 500 random alive animals and took their average value to be representative of all females. Though potentially not all owners of mares want to genotype their animals, the genotypes may be inferred, i.e. imputed, based on the mare's sire information and other relatives (Gengler et al. 2007; Sargolzaei et al. 2014). Alternatively, the average genomic relationship as well as allele frequencies at loci of interest may be inferred simply from the number of produced foals of genotyped sires. Carrier status, and thus genotype status, of loci causing e.g. dwarfism or distichiasis may also be inferred from phenotypic observations coupled with pedigree analysis. All in all, the beauty of the pseudo-female approach in OCS is that it only requires information for the male side and some assumptions about the female side. For completeness, it should be mentioned that the KFPS collects genotypes of all foals born since 2023. Once genotypes of all horses are available, these work-arounds will be irrelevant.

It should be noted that the observed pedigree inbreeding rate was also substantially higher than anticipated based on the set restriction. In the scenarios selecting against genetic conditions, the expectation of the inbreeding level in generation 10 is 12.22% $\left(F_{t}=1-{\left(1-\Delta F\right)}^{t}(1-F_{0})\right)$ since the inbreeding rate was restricted to 0.25% per generation and the average inbreeding level in generation 0 of the breeding program was 10%. We observed however an average pedigree inbreeding level of 13.5% (Fig. 15), which correspond to a realized inbreeding rate of 0.40% instead of 0.25% per generation. This discrepancy may be due to the stochasticity in the realized contributions of selected sires. Also, the random sample of 500 animals to be used as pseudo-females and to calculate the current average kinship may have resulted in a set with higher average relationship. Since the maximum allowed inbreeding level of the next generation was determined based on the current observed average kinship, this shift in base increases the allowed inbreeding level, and the errors accumulate over generations. Lastly, since only alive animals were considered to construct the pseudo-female, variation in lethality between families could have further increased the average kinship used when calculating the upper bound restriction for the average kinship in the next generation. The higher-than-anticipated pedigree inbreeding rate could have been avoided if the restrictions for inbreeding levels had been calculated in generation 0 for all future generations. Another practical approach may be to set a stricter inbreeding restriction than actually wanted. This is what we did here with setting 0.25% in OCS as the studbook actually aims for an inbreeding rate below 0.5% (Steensma et al. 2024).

In real life, the average inbreeding coefficient is about 17% in Friesians (Figure 3 of Steensma et al. (2024)) whereas our starting populations showed inbreeding coefficients of 10% on average (Fig. 15). We do not expect this discrepancy to affect the results because it only represents a shift in the mean. What could however lead to different results in real life is population structure or the occurrence of disorder alleles in only some families. Both would slow down purging of the variant. This is because de-selecting the same proportion of animals from all families does not change the average relationship whereas de-selecting entire families does. Fortunately for the Friesian breed, little to no population substructure has been found (Steensma et al. 2024).

### Using OCS in a breeding program with many stakeholders

Employing OCS in horse breeding is difficult, even with the pseudo-female approach. That is because of the many stakeholders involved and because of overlapping generations. It would be better to provide stallion owners with concrete guidelines informing them about the requirements to get their stallion approved. The following might help develop such requirements. We plotted all stallions that were preselected and considered by the OCS algorithm in a 3-dimensional plot (Fig. 16). The dimensions of the plot are the stallion's average relationship to the breed, its phenotype (here: withers height in cm), and the off-diagonal element of the $ZZ^{\prime }$ matrix for loci of interest pertaining to the stallion and the pseudo-female. Every stallion that is selected, i.e. has a non-zero contribution, is colored in pink. Based on the group of selected and non-selected individuals, a plane can be fitted that separates both groups. Since the coordinates for every new selection candidate are known (average relationship, phenotype, and average off-diagonal $ZZ^{\prime }$ score), a point can be added for that new stallion to check if it would be located in the group of individuals that OCS would select. Thus, it is possible to make (pre)selection decisions similar to those made by OCS without actually running OCS every time. The plane only needs to be updated every once in a while, for example, every year. For other populations, it may be advisable to recalculate the plane in shorter intervals if genetic gain is faster and the generation interval and/or the effective population size are smaller. It is possible to extend this strategy, e.g. to consider more dimensions. Informed by findings of Avendaño et al. (2004) presented in their Table 1 and their Fig. 2, the selective advantage of animals in breeding programs with diversity management may depend more on their Mendelian sampling term and less on their breeding value. The Mendelian sampling term is the deviation of performance of an individual from the average performance of its parents. We used phenotypes in this study, but any breeding program using genetic evaluations may choose to use breeding values or the estimated Mendelian sampling terms in a plot such as Fig. 16 to infer a good plane for group separation.

Using a plane just informs about whether a candidate is selected or not. Figure 16 also shows that animals further away from the plane get a larger contribution. To accommodate this, a selection index value could be derived by calculating a vector that is perpendicular to the plane. The weights of this vector can then be used as selection index weights for the respective dimensions. Instead of yes-no decisions, this allows to rank animals and assign contributions based on their selection index values. This then allows to use flexible mating quotas for stallions, i.e. the number of matings a stallion is allowed to have per year depends on this index value. This can be seen as a small modification to the stratified mating quotas proposed by Steensma et al. (2024) for the Friesian breed. Basing selection and contribution decisions on such perpendicular vector values would not result in exactly the same decisions as OCS, but in very similar ones. How close those OCS-like decisions are to real OCS decisions may differ among breeding programs.

### Importance of health vs lethality

We implicitly used the same selection index weights for all the 12 loci and the MHC region in this study. In real life, the importance of “color intensity” may be lower than that of e.g. “distichiasis” and so breeders may want to give different weights to different loci. This is possible with both the index-method and the matrix-method by multiplying the locus-specific Z or $ZZ^{\prime }$ matrices with the respective weight given to the locus. As shown, the matrix-method pulls stronger on high-frequency alleles when the target AF is 0% (Figs. 6 and 13). The matrix-method resulted in a slightly slower decrease of lethality than the index-method (Fig. 11) but caused a faster increase in genetically healthy horses (Fig. 12a). This is because genes that are not lethal but cause a genetic condition happen to have a higher AF in the Friesian horse population. This raises questions about the best objective of a breeding program. Should lethal alleles have the highest weight and be purged first? Or should the focus be on improving the health status, and thus welfare and veterinary costs, of horses that are born alive? Providing an answer is not in the scope of this study.

### Changing AF while keeping diversity in background

Simultaneously reducing the AF of IBH and managing genetic diversity in the same genomic region (MHC) is possible (Fig. 14). When MHC is not included, genetic diversity in the region decreases faster than if MHC is considered in selection decisions. Yet, even when considering MHC in selection decisions, genetic diversity initially depleted faster than in the control scenario (only inbreeding management). This may be avoided by simply placing a higher index weight on the MHC region. This is intuitive. The question to solve then is “how much weight?” which does not have an intuitive answer. A solution may be to remove the MHC region from the index (or the summed matrix) and instead fit diversity in the MHC region as an additional dimension in the OCS problem. Simply put, a relationship matrix only based on information of the MHC region can be fitted in the OCS problem as in Gómez-Romano et al. (2016). A restriction can then be imposed to make at most e.g. 0.5% inbreeding in the region (i.e. lose at most 0.5% heterozygosity). Setting a restriction of 0% inbreeding or even negative inbreeding, which corresponds to increasing heterozygosity, is possible as well. By fitting an extra GRM, a breeder can set more intuitive constrains which indirectly translate to placing a certain selection index weight. This approach led to satisfactory results with optiSel (not shown) but was left out of this study as the aim was to showcase the use of a combined index or matrix. If inbreeding control in the MHC region is possible, this then raises the question of why not to include the genomic region around the other loci in additional matrices. We propose that this should be standard practice to control the regional loss of diversity caused by strong selection on a linked locus.

We decided to use haplotypes spanning the entire 5 Mb MHC region as pseudo-alleles and thus treating the MHC region as a multi-allelic locus. This was straightforward but more refined strategies may be to consider e.g. 5 regions of each 1 Mb length, or to pair the haplotype-allele detection with pedigree analysis. Different approaches may be to set the desired AF of the genotyped SNPs to 50% to maximize heterozygosity, or to set desired AF to the AF of the founders in line with literature recommendations for constructing genomic relationships (Meuwissen et al. 2020; Gautason et al. 2023). The latter is difficult when founder AF are unknown, and while the former does indeed result in increased heterozygosity, this is only at the observed sites (results not shown). However, the more loci are genotyped in the region of interest, the better the approach of setting their desired frequency to 50% (based on increasing the number of genotyped SNPs in MHC region, not shown). This is simply because if (nearly) all loci are observed, they do not have to be managed indirectly through linkage on haplotypes. Determining the ideal approach to define alleles and what target frequencies to use was not the focus of this study. This is why we opted for an approach that observes and manages IBD so that identity by state at unobserved loci is indirectly managed (Powell et al. 2010). Our approach for the MHC region should not be considered ideal, but rather a proof of concept.

## Conclusion

We demonstrated that the average numerator matrix entry in VanRaden method 1 genomic relationship matrices equals 4 times the sum of squared AF deviations from the frequencies used for centering. This allows setting the centering frequencies to desired frequencies and optimizing genetic contributions to minimize for the average matrix entry to change allele frequencies (TAFT-OCS). This shifts the focus of selection from individuals to alleles. TAFT can be seen as a quadratic desired gains index for allele and haplotype frequencies. While the tested selection indices managed to purge unwanted alleles, only TAFT-OCS also managed to keep allele frequencies at predefined intermediate frequencies. Findings from application in the Friesian horse breed based on simulations with multiple deleterious alleles show that genetic improvement for a polygenic trait, purging of unwanted (deleterious) alleles and managing the rate of inbreeding can be done simultaneously. For example, within 6 generations time, the deleterious genetic conditions at 12 loci (with current allele frequencies ranging from 6 to 37%) could theoretically be effectively eliminated, while limiting the pedigree inbreeding rate to <0.5% and achieving considerable genetic gain for withers height (∼3.7 cm increase per generation). To ease implementation in real life, approximations are presented to use optimum-contribution-like selections in real life without explicitly calculating exact optimum contributions.

## Supplementary Material

iyag102_Supplementary_Data

## Data Availability

All data was simulated during the study. The R script for the toy selection scheme can be found on GitHub: https://github.com/tobiasniehoff/TAFT-Targeted_Allele_Frequency_Tuning. Otherwise, the authors state that the data necessary to confirm the conclusions presented in the article are represented fully within the article. Supplemental material available at GENETICS online.

## Appendix

The following provides proofs for the fact that the average matrix element of ZZ′ is identical to 4 times the sum over all loci of all squared AF deviations from the CAF. We worked out proofs in matrix notation and scalar notation because each notation may appeal to different readers.

### Proof in matrix notation

$$\bar{ZZ^{\prime }}=4\overset{p}{\underset{k=1}{\sum }}\;(AF_{k}-\mathrm{CA}F_{k}{)}^{2}$$

Let M be the genotype matrix with allele counts (size n×p), F be the p-length vector of centering allele frequencies, and $J_{n}$ an *n*-length vector of ones. $2J_{n}F^{\prime }$ is the P matrix used for centering allele counts in genomic relationship matrices. So, the matrix Z and the vector AF can be defined as follows:

$$Z=M-P=M-2J_{n}F^{\prime }$$

$$\mathrm{AF}=\frac{1}{2n}M^{\prime }J_{n}$$

Then the equation at the top can be verified by the following linear algebra manipulations (remember/note that ${J^{\prime }}_{n}J_{n}=n$, i.e. every element in $J_{n}$ is 1):

$$\begin{array}{rl} \bar{ZZ^{\prime }}\;= & \;\frac{1}{n^{2}}{J^{\prime }}_{n}(ZZ\prime )J_{n}\\ & ⇓\;\mathrm{Substitute}\;Z\\ = & \;\frac{1}{n^{2}}{J^{\prime }}_{n}(M-2J_{n}F^{\prime })(M-2J_{n}F^{\prime }{)}^{\prime }J_{n}\\ & ⇓\;\mathrm{Expand}\;\mathrm{the}\;\mathrm{product}.\\ = & \;\frac{1}{n^{2}}{J^{\prime }}_{n}(MM^{\prime }-2J_{n}F^{\prime }M^{\prime }-2MF{J^{\prime }}_{n}+4J_{n}F^{\prime }F{J^{\prime }}_{n})J_{n}\\ & ⇓\;\mathrm{Distribute}.\\ = & \;\frac{1}{n^{2}}{J^{\prime }}_{n}MM^{\prime }J_{n}-\frac{4}{n^{2}}{J^{\prime }}_{n}MF{J^{\prime }}_{n}J_{n}+\frac{4}{n^{2}}{J^{\prime }}_{n}J_{n}F^{\prime }F{J^{\prime }}_{n}J_{n}\\ & ⇓\;\mathrm{Use}\;{J^{\prime }}_{n}J_{n}=n\;\mathrm{and}\;\mathrm{simplify}.\\ = & \;\frac{1}{n^{2}}{J^{\prime }}_{n}MM^{\prime }J_{n}-\frac{4}{n}{J^{\prime }}_{n}MF+4F^{\prime }F\\ & ⇓\;\mathrm{Factor}\;\mathrm{out}\;4.\\ = & \;4\left(\frac{1}{4n^{2}}{J^{\prime }}_{n}MM^{\prime }J_{n}-\frac{1}{n}{J^{\prime }}_{n}MF+F^{\prime }F\right)\\ & ⇓\;\mathrm{Factor}\;\mathrm{the}\;\mathrm{quadratic}\;\mathrm{form}.\\ = & \;4{\left(\frac{1}{2n}M^{\prime }J_{n}-F\right)}^{\prime }\left(\frac{1}{2n}M^{\prime }J_{n}-F\right)\\ & ⇓\;\mathrm{Substitute}\;\mathrm{AF}=\frac{1}{2n}M^{\prime }J_{n}\;\mathrm{and}\;\mathrm{CAF}=F.\\ = & \;4(\mathrm{AF}-\mathrm{RAF}{)}^{\prime }(\mathrm{AF}-\mathrm{CAF})\\ & ⇓\;\mathrm{Write}\;\mathrm{the}\;\mathrm{final}\;\mathrm{result}\;\mathrm{as}\;a\;\mathrm{sum}\;\mathrm{over}\;\mathrm{all}\;p\;\mathrm{alleles}.\\ = & \;4\overset{p}{\underset{k=1}{\sum }}\;(AF_{k}-\mathrm{CA}F_{k}{)}^{2} \end{array}$$

To show that this also holds for the weighted average, e.g. when considering genetic contributions, consider that $\mathrm{AF}=0.5M^{\prime }c$, where c is a *n*-length vector that stores genetic contributions of candidates to the next generation. Thus, $0.5M^{\prime }c$ could also be used in the last substitution step.

### Proofs in scalar notation

#### Explanation for a particular matrix entry in $ZZ^{\prime }$

Z=M−P

Matrix element for position *ij* in matrix ${ZZ}^{\prime }$ is (animals are *i* and *j*):

$$(ZZ^{\prime }{)}_{i,j}=Z_{i}{Z^{\prime }}_{j}$$

$Z_{i,}$ and $Z_{\;j,}$ are the rows of the Z matrix belonging to animals *i* and *j*.

This can be written as a sum over all SNPs as below:

$(ZZ^{\prime }{)}_{i,j}=\overset{\mathrm{nsnp}}{\underset{k=1}{\sum }}\;Z_{i,k}Z_{\;j,k}$ , where $Z_{i,k}$ is the difference from the allele count to the centering frequency of individual *i* at SNP *k*.

Thus, it can be rewritten as follows:

$$\left(ZZ^{\prime }{)}_{i,j}=\overset{\mathrm{nsnp}}{\underset{k=1}{\sum }}\;Z_{i,k}Z_{\;j,k}=\overset{\mathrm{nsnp}}{\underset{k=1}{\sum }}\;(M_{i,k}-P_{,k})(M_{\;j,k}-P_{,k}\right)$$

where $M_{i,k}$ is the allele count (0,1,2) of individual *i* at SNP *k*. $P_{,k}$ is twice the centering frequency used for centering at SNP *k* ($CAF_{k}$). No animal needs to be specified for $P_{,k}$ as entries are identical for all animals in matrix P.

$M_{ik}$ and $P_{,k}$ can be expressed in terms of AF so that it can be written as follows:

$$\begin{array}{rl} (ZZ^{\prime }{)}_{i,j}= & \overset{\mathrm{nsnp}}{\underset{k=1}{\sum }}\;Z_{i,k}Z_{\;j,k}=\overset{\mathrm{nsnp}}{\underset{k=1}{\sum }}\;(M_{i,k}-P_{,k})(M_{\;j,k}-P_{,k})\\ = & \overset{\mathrm{nsnp}}{\underset{k=1}{\sum }}\;(2AF_{ik}-2\mathrm{CA}F_{k})(2AF_{\;jk}-2\mathrm{CA}F_{k})\\ = & \overset{\mathrm{nsnp}}{\underset{k=1}{\sum }}\;2(AF_{ik}-\mathrm{CA}F_{k})2(AF_{\;jk}-\mathrm{CA}F_{k})\\ = & \overset{\mathrm{nsnp}}{\underset{k=1}{\sum }}\;4(AF_{ik}-\mathrm{CA}F_{k})(AF_{\;jk}-\mathrm{CA}F_{k}) \end{array}$$

In words, this means that an element in $ZZ^{\prime }\;({\left(ZZ^{\prime }\right)}_{ij})$ is 4 times the sum of the products of the difference of AF from centering frequency of the 2 individuals. For elements on the diagonal, the self-relationship, the element $(ZZ^{\prime }{)}_{ii}$ is 4 times the sum of the squares of the differences of the allele frequencies of an individual to the centering frequencies.

#### Explanation for the average matrix entry in $ZZ^{\prime }$

In words, the average matrix entry $\left(\bar{ZZ^{\prime }}\right)$ is the average over all entries $\left({\left(ZZ^{\prime }\right)}_{ij}\right)$:

$$\bar{ZZ^{\prime }}=\frac{1}{n^{2}}\overset{n}{\underset{i=1}{\sum }}\;\overset{n}{\underset{\;j=1}{\sum }}\;(ZZ^{\prime }{)}_{i,j}=\frac{1}{n^{2}}\overset{n}{\underset{i=1}{\sum }}\;\overset{n}{\underset{\;j=1}{\sum }}\;\overset{\mathrm{nsnp}}{\underset{k=1}{\sum }}\;Z_{i,k}Z_{\;j,k}$$

The sum of entries in Z at a locus *k* over individuals can be written as follows:

$$\begin{array}{rl} \overset{n}{\underset{i=1}{\sum }}\;Z_{i,k}= & \overset{n}{\underset{i=1}{\sum }}\;(M_{i,k}-P_{,k})\\ = & \overset{n}{\underset{i=1}{\sum }}\;(2AF_{ik}-2\mathrm{CA}F_{k})\\ = & \left(\overset{n}{\underset{i=1}{\sum }}\;2AF_{ik}\right)-2nCAF_{k} \end{array}$$

(This last step uses that the sum of differences is the same as the difference of sums.)

$$\begin{array}{rl} & =2n{\bar{\mathrm{AF}}}_{k}-2\mathrm{nCA}F_{k}\\ & =2n({\bar{\mathrm{AF}}}_{k}-\mathrm{CA}F_{k}) \end{array}$$

Since ${\bar{\mathrm{AF}}}_{k}$ is the average AF over all animals, it is the same as the observed AF among all animals and can thus be written as $AF_{k}$.

$\overset{n}{\underset{i=1}{\sum }}\;\overset{n}{\underset{\;j=1}{\sum }}\;Z_{i,k}Z_{\;j,k}$ is an expansion of ${\left(\overset{n}{\underset{i}{\sum }}\;Z_{i,k}\right)}^{2}$:

$$\overset{n}{\underset{i=1}{\sum }}\;\overset{n}{\underset{\;j=1}{\sum }}\;Z_{i,k}Z_{\;j,k}=\left(\overset{n}{\underset{i}{\sum }}\;Z_{i,k}\right)\left(\overset{n}{\underset{i}{\sum }}\;Z_{\;j,k}\right)={\left(\overset{n}{\underset{i}{\sum }}\;Z_{i,k}\right)}^{2}$$

${\left(\overset{n}{\underset{i}{\sum }}\;Z_{i,k}\right)}^{2}$ can be written in terms of allele frequencies and centering allele frequencies:

$${\left(\overset{n}{\underset{i}{\sum }}\;Z_{i,k}\right)}^{2}=(2n(AF_{k}-\mathrm{CA}F_{k}){)}^{2}=4n^{2}(AF_{k}-\mathrm{CA}F_{k}{)}^{2}$$

Thus, we can write the average element of $ZZ^{\prime }$ as follows:

$$\begin{array}{rl} \bar{ZZ^{\prime }}\;= & \;\frac{1}{n^{2}}\overset{n}{\underset{i=1}{\sum }}\;\overset{n}{\underset{\;j=1}{\sum }}\;(ZZ^{\prime }{)}_{i,j}=\;\frac{1}{n^{2}}\overset{\mathrm{nsnp}}{\underset{k=1}{\sum }}\;\overset{n}{\underset{i=1}{\sum }}\;\overset{n}{\underset{\;j=1}{\sum }}\;Z_{i,k}Z_{\;j,k}=\;\frac{1}{n^{2}}\overset{\mathrm{nsnp}}{\underset{k=1}{\sum }}\;{\left(\overset{n}{\underset{i}{\sum }}\;Z_{i,k}\right)}^{2}\\ = & \;\frac{1}{n^{2}}\overset{\mathrm{nsnp}}{\underset{k=1}{\sum }}\;4n^{2}(AF_{k}-\mathrm{CA}F_{k}{)}^{2}\\ = & \overset{\mathrm{nsnp}}{\underset{k=1}{\sum }}\;4(AF_{k}-\mathrm{CA}F_{k}{)}^{2}\\ = & \;4\overset{\mathrm{nsnp}}{\underset{k=1}{\sum }}\;(AF_{k}-\mathrm{CA}F_{k}{)}^{2} \end{array}$$

#### Extension to proof for average matrix entry in $ZZ^{\prime }$ weighted by contributions c

The above is a proof to show that the average element in ${ZZ}^{\prime }$ is identical to $4\overset{nsnp}{\underset{k=1}{\sum }}\;(AF_{k}-CAF_{k}{)}^{2}$. The following is an extension to proof that the same applies to ${ZZ}^{\prime }$ that is weighted by contributions, i.e. $c^{\prime }(ZZ^{\prime })c$. c is a vector that stores the genetic contribution of an animal to the next generation. Note that when calculating the average element of ${ZZ}^{\prime }$ above, every value in c is implicitly 1/n.

Here, same as above but rewritten so that every element is directly weighted instead of weighing the final sum:

$$\begin{array}{rl} \bar{ZZ^{\prime }}\;= & \;\frac{1}{n^{2}}\overset{n}{\underset{i=1}{\sum }}\;\overset{n}{\underset{\;j=1}{\sum }}\;(ZZ^{\prime }{)}_{i,j}=\frac{1}{n^{2}}\overset{n}{\underset{i=1}{\sum }}\;\overset{n}{\underset{\;j=1}{\sum }}\;\overset{\mathrm{nsnp}}{\underset{k=1}{\sum }}\;Z_{i,k}Z_{\;j,k}\\ = & \overset{n}{\underset{i=1}{\sum }}\;\overset{n}{\underset{\;j=1}{\sum }}\frac{1}{n^{2}}(ZZ^{\prime }{)}_{i,j}=\overset{n}{\underset{i=1}{\sum }}\;\overset{n}{\underset{\;j=1}{\sum }}\;\overset{\mathrm{nsnp}}{\underset{k=1}{\sum }}\;\left(\frac{1}{n}Z_{i,k}\right)\left(\frac{1}{n}Z_{\;j,k}\right) \end{array}$$

Same as before but rewritten so that the contribution per animal can vary according to c (above, the contribution was 1/*n* for all):

$$c^{\prime }(ZZ^{\prime })c=\overset{n}{\underset{i=1}{\sum }}\;\overset{n}{\underset{\;j=1}{\sum }}\;{c^{\prime }}_{i}(ZZ^{\prime }{)}_{i,j}c_{j}=\overset{n}{\underset{i=1}{\sum }}\;\overset{n}{\underset{\;j=1}{\sum }}\;\overset{\mathrm{nsnp}}{\underset{k=1}{\sum }}\;(c_{i}Z_{i,k})(c_{j}Z_{\;j,k})$$

The sum of entries in Z at a locus *k* weighted by contributions c over individuals can be written as follows. Note that $\overset{n}{\underset{i=1}{\sum }}\;c_{i}=1$ and $\frac{M_{i,k}}{2}=AF_{ik}$. $\overset{n}{\underset{i=1}{\sum }}\;c_{i}AF_{ik}=AF_{k,t+1}$, i.e. the sum of contribution-weighted allele frequencies of individuals, is the AF in the next generation (t+1) (if animals contribute according to c):

$$\begin{array}{rl} \overset{n}{\underset{i=1}{\sum }}\;c_{i}Z_{i,k}= & \overset{n}{\underset{i=1}{\sum }}\;c_{i}(M_{i,k}-P_{,k})\\ = & \overset{n}{\underset{i=1}{\sum }}\;c_{i}(2AF_{ik}-2\mathrm{CA}F_{k})\\ = & \left(\overset{n}{\underset{i=1}{\sum }}\;c_{i}2AF_{ik}\right)-\left(\overset{n}{\underset{i=1}{\sum }}\;c_{i}2\mathrm{CA}F_{k}\right)\\ = & \left(2\overset{n}{\underset{i=1}{\sum }}\;c_{i}AF_{ik}\right)-2\mathrm{CA}F_{k}\\ = & 2AF_{k,t+1}-2\mathrm{CA}F_{k}=2(AF_{k,t+1}-\mathrm{CA}F_{k}) \end{array}$$

This can be inserted and simplified:

$$\begin{array}{rl} c^{\prime }(ZZ^{\prime })c\;= & \overset{n}{\underset{i=1}{\sum }}\;\overset{n}{\underset{\;j=1}{\sum }}\;\overset{\mathrm{nsnp}}{\underset{k=1}{\sum }}\;(c_{i}Z_{i,k})(c_{j}Z_{\;j,k})\\ = & \overset{\mathrm{nsnp}}{\underset{k=1}{\sum }}\;(2(AF_{k,t+1}-\mathrm{RA}F_{k}))(2(AF_{k,t+1}-\mathrm{CA}F_{k}))\\ = & \overset{\mathrm{nsnp}}{\underset{k=1}{\sum }}\;(2(AF_{k,t+1}-\mathrm{CA}F_{k}){)}^{2}\\ = & \overset{\mathrm{nsnp}}{\underset{k=1}{\sum }}\;4(AF_{k,t+1}-\mathrm{CA}F_{k}{)}^{2} \end{array}$$
