# Supplementary material for: Targeted allele frequency tuning in breeding populations via alternative optimum contribution selection
Source: Genetics. 2026 Apr 24;233(3):iyag102. doi: 10.1093/genetics/iyag102 (PMC13334115; doi:10.1093/genetics/iyag102)
Supplement: iyag102_Supplementary_Data [file iyag102_supplementary_data.docx]

| Supplementary Table ST1: Historic effective population size in any historic generation considered by coalescence simulation in runMacs2() of the AlphaSimR package (Gaynor et al., 2021). | |
| --- | --- |
| Historic effective population size (Ne) | Historic generation |
| 62,000 | 33,154^*^ |
| 17,000 | 3,354^*^ |
| 10,000 | 2,354^*^ |
| 7,000 | 1,754^*^ |
| 3,500 | 654^*^ |
| 2,500 | 454^*^ |
| 2,000 | 154^*^ |
| 1,500 | 24^*^ |
| 200 | 20 |
| 100 | 10^+^ |
| 70 | 9^+^ |
| 50 | 8^+^ |
| 25 | 7^+^ |
| 25 | 6^+^ |
| 35 | 5^+^ |
| 50 | 4^+^ |
| 50 | 3^+^ |
| 50 | 2^+^ |
| 80 | 1^+^ |
| ^*^ suggested by runMacs2() for cattle (<https://rdrr.io/cran/AlphaSimR/man/runMacs2.html>) based on cattle demography following MacLeod et al. (2013)  ^+^ run in MoBPS to build up pedigree recording starting from generation 10, resembling Friesian horse population (Steensma et al., 2024) | |

Gaynor, R. C., G. Gorjanc, and J. M. Hickey. 2021. AlphaSimR: an R package for breeding program simulations. G3 (Bethesda) 11: jkaa017.

MacLeod, I. M., D. M. Larkin, H. A. Lewin, B. J. Hayes, and M. E. Goddard. 2013. Inferring Demography from Runs of Homozygosity in Whole-Genome Sequence, with Correction for Sequence Errors. Mol. Biol. Evol. 30: 2209-2223.

Steensma, M. J., H. P. Doekes, T. Pook, M. F. L. Derks, N. Bakker, and B. J. Ducro. 2024. Evaluation of breeding strategies to reduce the inbreeding rate in the Friesian horse population: Looking back and moving forward. J. Anim. Breed. Genet. 141: 668-684.
